# Mapping the landscape of tandem repeat variability by targeted long read single molecule sequencing in familial X-linked intellectual disability

**DOI:** 10.1186/s12920-018-0446-7

**Published:** 2018-12-19

**Authors:** Alena Zablotskaya, Hilde Van Esch, Kevin J. Verstrepen, Guy Froyen, Joris R. Vermeesch

**Affiliations:** 1 Department of Human Genetics and Center for Human Genetics, Laboratory for Cytogenetics and Genome Research, University Hospitals Leuven, KU Leuven, O&N I Herestraat 49 - box 606, 3000 Leuven, Belgium; 2 Department of Human Genetics and Center for Human Genetics, Laboratory for Genetics of Cognition, University Hospitals Leuven, KU Leuven, O&N I Herestraat 49 - box 606, 3000 Leuven, Belgium; 3 VIB Center for Microbiology and CMPG Lab for Genetics and Genomics, KU Leuven, Gaston Geenslaan 1 - box 2471, 3001 Leuven, Belgium; 4 Clinical Biology, Laboratory for Molecular Diagnostics, Jessa Hospital, Stadsomvaart 11, 3500 Hasselt, Belgium

**Keywords:** Tandem repeats, Expansion, Single molecule sequencing, X-linked intellectual disability

## Abstract

**Background:**

The etiology of more than half of all patients with X-linked intellectual disability remains elusive, despite array-based comparative genomic hybridization, whole exome or genome sequencing. Since short read massive parallel sequencing approaches do not allow the detection of larger tandem repeat expansions, we hypothesized that such expansions could be a hidden cause of X-linked intellectual disability.

**Methods:**

We selectively captured over 1800 tandem repeats on the X chromosome and characterized them by long read single molecule sequencing in 3 families with idiopathic X-linked intellectual disability.

**Results:**

In male DNA samples, full tandem repeat length sequences were obtained for 88–93% of the targets and up to 99.6% of the repeats with a moderate guanine-cytosine content. Read length and analysis pipeline allow to detect cases of > 900 bp tandem repeat expansion. In one family, one repeat expansion co-occurs with down-regulation of the neighboring *MIR222* gene. This gene has previously been implicated in intellectual disability and is apparently linked to *FMR1* and *NEFH* overexpression associated with neurological disorders.

**Conclusions:**

This study demonstrates the power of single molecule sequencing to measure tandem repeat lengths and detect expansions, and suggests that tandem repeat mutations may be a hidden cause of X-linked intellectual disability.

**Electronic supplementary material:**

The online version of this article (10.1186/s12920-018-0446-7) contains supplementary material, which is available to authorized users.

## Background

Intellectual disability (ID) has a prevalence of 2.3%, making it a prime socio-economical problem [1]. ID is a very complex and heterogeneous disorder that can be caused by genetic factors, environmental factors or a combination of both. As a result, the etiology remains unknown in ~ 30% of cases. X chromosome-linked ID (XLID) has served as a model for the genetics underlying ID, in part because it is approximately 30% more prevalent in males than in females, suggesting that important causative genetic loci are located on the X-chromosome [2].

In the last 15 years, candidate gene mutation screening [3, 4], hybridization-based array screens [5, 6] including high resolution array-CGH [7–10], and massively-parallel sequencing (MPS) screens [11–14] led to the identification of many genes associated with ID. It became clear that genetic causes of ID are highly heterogeneous, as the reported mutations explain only a small number of ID families [2]. For example, a Sanger sequencing-based screen of all exons on the X-chromosome in 208 XLID families only revealed causal mutations in 25% of families [15]. Later, MPS allowed for much higher throughput identification of disease-associated mutations, deletions and duplications. However, this groundbreaking method could not resolve more than 20% of the remaining cases, as illustrated by an X chromosome-specific exome MPS screen in 405 XLID families [14]. Thus, despite the large number of studies and significant technological progress, the etiology of ID remains unsolved in at least 40% of XLID families.

These figures strongly suggest that the missing mutations should be searched for in the non-coding regions of the X chromosome, or in regions that currently escape analysis. An intergenic variant identified by targeted MPS on the complete linkage interval of a large XLID family has been associated with enhanced expression of *HCFC1* in a family with nonsyndromic ID [16] demonstrating that indeed regulatory mutations contribute to ID.

Another group of regions that have been neglected are the tandem repeats. Tandem repeats largely escape mutation analysis because their larger sizes are not covered by short read sequencing technologies. In addition, the sequence reads often fail to be mapped back to the reference genome due to their repetitive nature.

Tandem repeats are DNA sequences consisting of multiple (almost) identical copies of a short (typically 1–50 nt) unit sequence that is repeated in a head-to-tail manner. Such repeats are arbitrarily divided into microsatellites and minisatellites, depending on repeat unit length. Tandem repeats are abundantly present in the human genome including the coding sequences and promoters [17], but thorough variation analysis is lacking due to technical challenges. The mutation rate of repeat regions is typically at least one order of magnitude higher than those in non-repetitive DNA, and as a consequence, variation in repeat length in coding or regulatory regions has a high probability to influence the function or expression of genes [18–20]. Despite all those features, repeats are often overlooked as prime targets for disease-related mutations. Moreover, the most commonly used MPS instruments from Illumina and Thermofisher provide average read lengths of 150–200 nt, which is too short to read through most repeats. Even paired-end sequencing does not increase read length in this case, because in order to obtain reliable sequences both reads of a pair should span a full tandem repeat with flanks. Therefore, long-read sequencing technologies are more suitable to study repeat variations. Recently, single molecule real-time sequencing has been introduced to study tandem repeats through long-range PCR amplicons spanning a single repeat of interest. Despite the significant error rate of this newest MPS technology, an accurate consensus tandem repeat can be reconstructed via a local de novo assembly [21–23]. In addition, this long read MPS platform is especially valuable to study expansions because of the circular nature of the reads. Multiple passes through the read sequence allow to generate a consensus sequence which facilitates discrimination between sequencing errors and PCR artefacts (“stutters”), that are commonly obscuring tandem repeat analyses.

To test the hypothesis that tandem repeat expansions are a hidden cause of XLID, we set out to selectively target repetitive sequences on the X chromosome and characterize them by single molecule sequencing using the PacBio platform. Specifically, we targeted more than 1800 tandem repeats on the X-chromosome in 3 families with idiopathic X-linked intellectual disability in whom previous methods did not detect any potential genetic cause. Our analysis identified one candidate causal repeat expansion in one family. Gene expression analysis showed down-regulation of the neighboring *MIR222* and, indirectly, *FMR1* and *NEFH* overexpression. This study suggests that tandem repeat mutations may be a hidden cause of XLID and potentially of other diseases as well.

## Methods

### Selection of tandem repeats and capture probe design

A list of tandem repeats on the X chromosome was obtained from the hg19 human reference genome (UCSC [24]) with the ETANDEM tool (EMBOSS package [25]) and was complemented by repeats from several other sources [19, 26–29] as described by Duitama et al. [30] bringing the total number of target repeats to 43,106. They included repetitive loci with a unit size 1–50 bp, copy numbers of 2–809, full length 22–4048 bp, and GC content including the extreme values of 0 and 100%.

All tandem repeats were annotated according to their position relative to a gene and divided into two groups: presumably functional (i.e. located in coding and regulatory regions) and likely non-functional as previously described [30]. The variability potential of these repeats was predicted by the SERV score [17] based on the following characteristics: unit length, copy number in the reference genome, and intralocus homology. SERV values 1–3 correspond to the highly variable tandem repeats that are usually used for genotyping.

We aimed to sequence around 2000 repeat loci on the X chromosome. We reasoned that a total of at least 500 flanking base pairs should be kept in a 1 kb consensus sequence for probe annealing sites and repeat variation, hence the maximal length threshold of 500 bp for tandem repeats. We also kept a number of intronic and intergenic tandem repeats on the X chromosome to narrow down the linkage intervals with inheritance patterns of more than 2 haplotype specific alleles per repetitive locus.

First, tandem repeats were pre-selected based on their characteristics, presumed functionality and/ or predicted degree of polymorphism (Table 1): 1) 353 tandem repeats in coding regions with the total size up to 500 bp; 2) 174 tandem repeats with SERV score ≥ 1 and total length ≤ 500 bp, which are located in regulatory sites (CpG islands, transcription factor binding sites, regions upstream or downstream from a gene, including micro-RNA genes); 3) 390 regulatory repeats of any size with SERV scores 0.4–1; 4) 68 regulatory tandem repeats within 1000 bp distance from the 112 genes that are known to be involved in XLID (Greenwood Genetic Center, [31]) and not yet included for probe design; 5) 1000 non-functional tandem repeats evenly distributed over the X chromosome with SERV score > 0.8, at least 15 copies in the reference genome, unit length > 1 bp, total length ≤ 500 bp, GC content 30–70%, and at least 3 different capture probes available.

**Table 1:** Initial selection of possible targets for subsequent probe design, and final selection of tandem repeats for capture and sequencing

#	Selection groups	Predicted variability	Total repeat length	Unit length	Copy number	*N*	Results of modified probe design (final selection)							
							1 probes	2 probes	3 probes	4 probes	All targeted		Not included	
1	Coding repeats	Any SERV	Any total length	Any unit	Any copy num.	368					305	82,88%	63	17,12%
			Repeat length ≤ 500 bp			353	36	37	74	158				
2	Regulatory repeats (top variability)	SERV ≥1	Any total length	Any unit	Any copy num.	181					149	82,32%	32	17,68%
			Repeat length ≤ 500 bp			174	23	36	78	12				
3	Regulatory repeats (lower variability)	0,4 < SERV < 1	Repeat length ≤ 520 bp (all)	Any unit	Any copy num.	390	62	55	100	101	318	81,54%	72	18,46%
4	Additional regulatory repeats within 1 kb from the genes involved in XLID (not yet included in groups 2–3)	Any SERV (−0,92 – 0,37)	Any total length (< 250 bp)	Any unit	Any copy num.	68	2	5	19	39	65	95,59%	3	4,41%
	Total (‘functional’)					1007	123	133	271	310	837	83,12%	170	>16,88%
5	Intronic repeats only	SERV > 0,8	Repeat length ≤ 500 bp	Unit ≥2 bp	≥ 15 copies	3431	filtered out	filtered out	516	24	540	15,74%	2891	84,26%
6	Intergenic repeats only	SERV > 1	Repeat length ≤ 500 bp	Unit ≥2 bp	≥ 15 copies	4126			440	20	460	11,15%	3666	88,85%
	Total (‘unknown significance’)					7557			956	44	1000	>13,23%	6557	86,77%
	Total (all)					8564>	123	133	1227	354	1837	21,45	6727	78,55%

SERV≥1 corresponds to the high predicted variability

Then probe design was performed as previously described [30] and included left and right flanking probes, spanning probes centered on tandem repeats, and double probes containing both flanks of a target. In total, 9969 probes generated for 4503 tandem repeats (715 functional and 3788 non-functional repeats) matched uniqueness criterion, i.e. were not predicted to hybridize aspecifically (Table S1). Subsequently, for those 270 repeats where it was not possible to generate unique capture probes, another round of probe design was performed with modified settings: flanking probes and two parts of a double probe were allowed to shift outwards from a repeat by up to 500 bp, and the most proximal available unique probe was chosen for the application (see Additional file 1). This approach allowed us to generate 118 new probes and add 66 tandem repeats to our final repeat selection. To increase the total number of available probes from 1 to 3 or 4, the same strategy was applied to the 7 ‘XLID repeats’ and was successful for 6 of them.

Finally, we examined the distribution of 204 functional repeats which could not be targeted after the above described steps. These repeats were tested for homology to other genomic loci using the Bowtie alignment tool [32] with the following settings: -e 200 -n 3 -y -l 15 -k 10 (or -k 30, depending on the expected number of locus-specific alignments). When a repeat together with its capture probes revealed a local specificity, but no homology to unrelated regions (e.g. showed homology only with other sequences within a cluster of a locally duplicated region), this repeat was kept in the final selection.

Following this approach, two distinguishable clusters of untargeted repeats were found at both ends of the X chromosome. Eighteen tandem repeats on the p arm fall in the pseudoautosomal region 1 (PAR1) with a 100% homology with the corresponding region on the Y chromosome. For 14 out of 18 repeats in PAR1 there was no high homology detected other than with the Y chromosome, and 1 to 3 locus-specific probes were available per repeat. Similarly, we reanalyzed probes for 13 repeats in PAR2 on the q arm, but no locus-specific probes were found due to a high similarity of these sequences to other regions on the chromosomes X, Y or autosomes.

Two other clusters of so far untargeted tandem repeats were detected on the q arm of the X chromosome. A total of 23 presumably regulatory repeats, located in CpG islands, build up a cluster at Xq23. They belong to a 53 kb region (chrX:114,952,840-115,006,118) with a complex structure, which reveals a number of local duplications. For this reason, all the fragments of the original probes were realigned to the reference genome to scrutinize more top alignments. As a result, 3 probes became available for each repeat within the cluster, which cross-align to repeats within this region, but not to other positions in the genome. All 23 repeats were added to the final selection of tandem repeats.

A cluster with 15 untargeted coding repeats represents members of the cancer/testis gene family 47, also known as CT47. It is comprised of 13 nearly identical loci clustered in a 118 kb region at Xq24 (chrX:120,002,680-120,120,440). Following the same approach, we enriched our final selection of tandem repeats with 11 loci, each having 3 cluster-specific probes.

This approach resulted in 1837 tandem repeats. They contain 837 (83%) presumably functional repeats on the X chromosome (Table 1) including repeats implicated in spinal bulbar muscular atrophy, fragile X and Fragile X E syndromes.

All probes obtained for tandem repeat capture were replicated based on the available types of probes and their GC content, as described by Duitama et al. [30] (Additional file 3: Figure S1), except that each probe with a GC content below 40% or above 70% was multiplied by 4. The resulting probe design contains 21,386 probes with a total capture size of 0.49 Mb and is given in the Additional file 2. It includes all the necessary information for ordering the SureSelect probes (Sheet 1), and a full description of the available probes for each target including their genomic positions, replication numbers and efficiency of a particular probe combination (Sheet 2). Each tandem repeat in the final selection is targeted by 4 to 20 probes of 1 to 4 types (Additional file 3:Figure S1).

### Selection of XLID cases

**Fig. 1:**
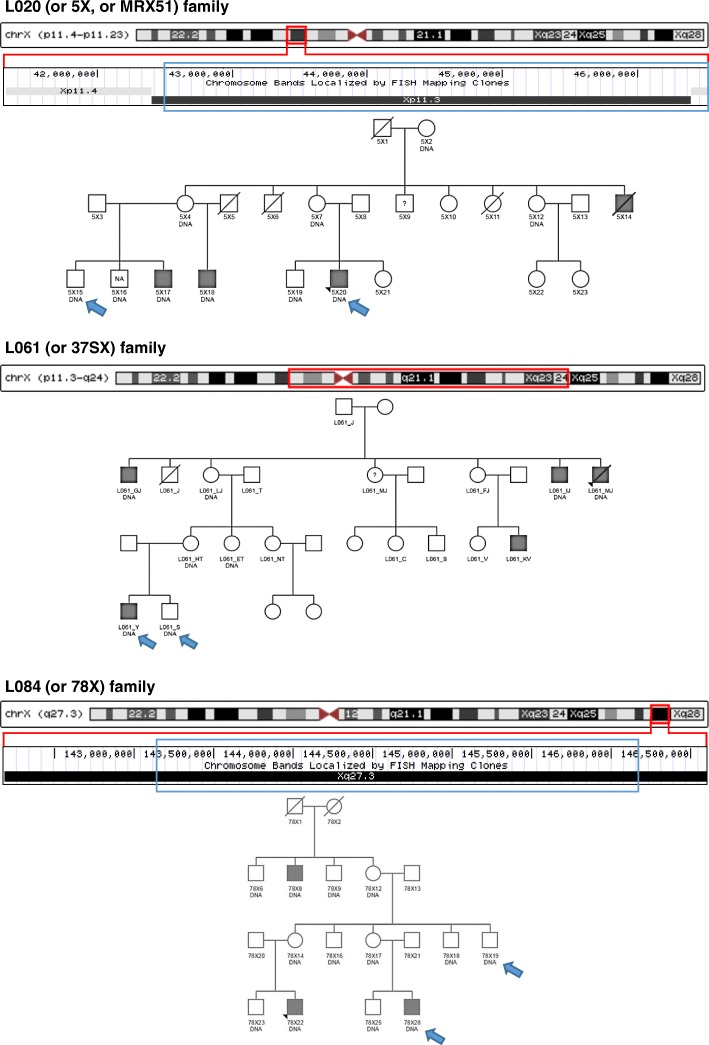
Pedigrees of the selected families with idiopathic XLID. Probands are marked with a black arrow head. Grey filling indicates ID phenotype. Blue arrows point out individuals selected for targeted capture and sequencing of tandem repeats. ‘DNA’ stands for available genetic material. Ideogram of the X-chromosome with a zoom into the linkage interval is depicted for each family. Red and blue boxes indicate initial and refined linkage intervals, correspondingly

Initially, we selected familial cases of ID recorded by the University Hospital of Leuven where consecutive studies, such as karyotyping, individual screening of known ID genes [4], X-array-CGH [8], or X-exome sequencing [14] did not reveal any clear pathogenic variants. The protocol was approved by the appropriate Institutional Review Board of the University Hospital of Leuven, Belgium, and informed consent was obtained from the parents of the affected patients and their healthy family members. In these families, we first checked for autosomal linkage with Merlin and for X-chromosomal segregation with Minx with default parameters (MERLIN package) using available STR profiles of the family members. For several families, SNP-arrays were additionally performed to confirm or disprove X-linkage. The HumanCytoSNP-12v2.1 BeadChips (Illumina) were prepared according to manufacturer’s instructions, and their results were analyzed with Merlin. In two families, idiopathic ID was confirmed to be X-linked: L020 (or 5X, or MRX51) [33], L061 (or 37SX). For one more family, L084 (or 78X), a suggestive linkage interval on the X was obtained. In each family, an EBV-PBL cell line from the proband was available. Per family, DNA from an affected and an unaffected male were chosen for sequencing of the X-chromosomal tandem repeats: 5X20 and 5X15; 78X28 and 78X19; L061_Y and L061_S (Fig. 1). Genomic DNA samples were provided by the DNA biobank of the University Hospital of Leuven.

The affected individuals of the L020 family all present with non-syndromal ID ranging from mild to moderate as described by Claes et al. [33]. It is important to note that the 5X9 member of the L020 family was treated as affected, and 5X16 as unaffected. The affected individuals of the L061 family presented with non-syndromal moderate ID. Two individuals also presented with epilepsy. They were non-dysmorphic and had normal neurological examination except for individual L061_MJ, who experienced a cerebrovascular accident at the age of 47 years. Family L084 includes 3 affected males with mild to moderate non-syndromal ID. The youngest individual also presented with spastic paraplegia starting in young adulthood.

### Library preparation and sequencing

DNA samples were sonicated in a Focused-ultrasonicator (Covaris) into fragments with an average size of 800–1000 bp. Library preparation was done following the manufacturer’s instructions (SureSelect Target Enrichment System for Roche 454 GS FLX and GS Junior Sequencing Platforms). SureSelect libraries were directed for SMRTbell library preparation (2 kb Template Preparation Procedure: DNA damage repair till first purification of SMRTbell templates using 0.6X AMPure PB beads) and sequenced on the Pacific Biosciences RS II machine with the P5-C3 or P6-C4 reagent kit. Each library was run on three SMRT cells. Sequencing was performed by the Genomics Core of the University Hospital of Leuven. Fastq files were obtained with a minimal requirement of 6 subreads per read of insert.

### Analysis of the sequencing data

We developed a bioinformatical pipeline for retrieving information on the targeted tandem repeats from the sequencing data and their subsequent genotyping (Additional file 3: Figure S2). First, adaptor sequences were removed by trimming 32 nucleotides at both sides of a read (see Additional file 4). Alignment of the trimmed reads to the reference genome was performed using Burrows-Wheeler Aligner and Smith-Waterman alignment algorithm (BWA SW) [34]. PCR duplicates were removed with the MarkDuplicates tool (Picard tools package [35]). BEDtools [36] were used to obtain the aligned reads, which were mapped within 1000 bases from the target sites, intersected the targeted tandem repeats, or spanned full tandem repeats. A custom script (see Additional files 5 and 6) was used to estimate the number of reads spanning tandem repeats together with their 20 nt-long flanking regions, considering soft-clipped reads with several partial alignments to the flanks.

Reads of insert were further analyzed with the TSSV tool [37] with a -d option, but before that an additional step was introduced to increase specificity and speed up the analysis. For each tandem repeat a custom script (see Additional file 7) filtered those reads that were mapped within 300 bases distance from the targeted region, and created separate input files for the TSSV tool: a fasta file with filtered reads and a corresponding TSSV library. TSSV libraries included names of tandem repeats, left and right flanking sequences which were fetched from the reference genome on the Galaxy platform, and repetitive units with expected ranges of copy number (lowest and highest copy numbers set equal to the reference copy number in this case). For tandem repeats included in clusters at Xq23 and Xq24, unique cluster representatives were searched for in fasta files containing all reads mapped to the X chromosome.

TSSV output was processed with a custom script (see Additional file 8), which analyzes allele lengths, calculates copy numbers, implements genotyping principles described in Duitama et al. [30], and determines which copy numbers correspond to partial reads where only one of the flanking regions was found by the TSSV.

### Validation of sequencing derived genotypes and their inheritance in a family

The genotypes obtained by massively parallel sequencing and data analysis were validated by fragment analysis in family members and if necessary in up to 100 controls. Control sampling comprised unaffected members of other families and patients admitted to the hospital with other, non-neurological diseases. PCR was performed in two rounds consisting of 15 and 20 cycles respectively. The first round was performed on 50 ng genomic DNA in a 25 μl mixture using Taq DNA Polymerase (Invitrogen) and 0.2 μM unlabeled specific primers designed with Primer3 [38]. All forward primers contained a 21 bp extension of the M13 sequence at the 5′-end. Of the first PCR product, 2 μl were used as a template for the second reaction containing a FAM-labelled M13 primer and a locus-specific reverse primer. Final products were run on an ABI3500xL Genetic Analyzer (Thermofisher) with the GeneScan 500 ROX Size Standard (Thermofisher). Fragment lengths were analyzed with the GeneMapper v4.1 software (Thermofisher). For the loci possibly expanded and located within a linkage interval, we performed Sanger sequencing on the first PCR products with the respective unlabeled primers and the BigDye v3.1 cycle sequencing kit. Products were analyzed on the ABI3500xL, and resulting sequences were aligned with the BioEdit v7.1 software (Ibis Biosciences) to count the exact number of units in a tandem repeat. PCR primers used in this study are given in Additional file 9.

### Quantitative PCR

For *MIR222* expression analysis RNA was extracted from EBV-PBL cell lines using mirVana miRNA Isolation Kit (Thermofisher) following small RNA enrichment procedure. RT-PCR for small RNA was performed using the TaqMan MicroRNA Reverse Transcription Kit (Thermofisher). Expression level was measured by qPCR using miRNA-specific TaqMan Small RNA Assays (Thermofisher) with 2 endogenous control miRNAs: *hsa-let-7f-5p*, *MIR98*. This was done in two independent RT-qPCR experiments. For expression analysis of other genes, total RNA was extracted from the non-confluent cell cultures using RNeasy Mini Kit (Qiagen), and cDNA was synthesized with Superscript Reverse Transcriptase and random primers (Thermofisher). Expression levels were measured 2–3 times by qPCR using SYBR Green on the LC480 apparatus (Roche) with 3 endogenous control genes: *GUSB*, *HPRT1*, *PORCN*. Primers used for qPCR in this study are given in Table S2, Additional file 3.

### Total RNA sequencing

Total RNA was extracted from the non-confluent EBV-PBL cell cultures using RNeasy Mini Kit (Qiagen), and cDNA was synthesized with Superscript Reverse Transcriptase and random primers (Thermofisher). Total RNA single-end Illumina sequencing generated 50 bp reads, which were mapped to the hg19 human reference genome using Tophat version 2.0.6 [39]. BAM files were handled with SAMtools version 0.1.18 [40]. Quantification of reads per gene and differential expression analysis was performed with Cufflinks version 2.0.2 [41]. Differentially expressed genes were first pre-selected with the false discovery rate of 5%. To filter the most deregulated genes, they were ranked according to the ratio (R) of the difference between the patient expression value (P) and the closest control value (C) to expression range within controls: R = |P-C|/(C_max_-C_min_). Loci with statistically significant difference of expression (*p* < 0.001) in patient comparing to three controls were subjected to pathway enrichment analysis using IPA (Qiagen).

## Results

### Tandem repeat capture and sequencing in XLID families

We analyzed three families with idiopathic XLID for X-chromosomal tandem repeat variation. In the past, in families L020, L061 and L084 neither full coverage X chromosomal microarrays, nor X-exome sequencing [4, 14] revealed any pathogenic variants. In those families, linkage analysis results in LOD scores of respectively 2.406, 2.23 and 0.932, suggesting X-linkage (see Materials and Methods). The family trees, affected and unaffected family members selected for targeted resequencing, as well as the linkage intervals are shown in Fig. 1 and Table 2. Taken that the average prevalence of intellectual disability in Western countries is 2%, and the majority of cases are sporadic, we could use this frequency to estimate the probability of at least two or three causal factors (de novo mutations and/or environmental factors) co-occurring within one family: 4 × 10^− 4^–8 × 10^− 6^. Therefore, we consider a combination of several different etiologies within a single family to be unlikely, especially when the detected linkage interval on the X chromosome is significant (LOD > 2).

**Table 2:** Linkage analysis confirmed X-linkage in 3 familial cases of intellectual disability

Family	Linkage interval		Mbp	LOD
L020 (or 5X, or MRX51)	Initial	chrX:41,323,975-46,534,411	5.21	2.406
	Refined	chrX:42,505,938-46,534,357	4.03	2.41
L061 (or 37SX)	Initial	chrX:46,179,305-103,255,350	57.08	2.23
		chrX:103,255,350-112,506,789	9.25	2.23
		chrX:112,516,866-120,180,324	7.66	2.23
L084 (or 78X)	Initial	chrX:142,184,383-146,607,898	4.42	0.932
	Refined	chrX:143,125,342-146,175,617	3.05	1.042

For each XLID family an affected and an unaffected male was selected for targeted capture and long-read single-molecule sequencing of the tandem repeats on the X-chromosome (Fig. 1). On average, more than 135,000 reads are obtained per sample, of which 28% map within 1 kb from our targets, and almost 20% of the reads are useful for genotyping, as they span a target together with both flanks (Table 3). For 8.68% of the targets we could not obtain any reads, and for 1.61% of the loci we only obtained sequences that do not span the full repeat length. We obtained full sequences for 88 to 93% of the targets in the sequenced individuals with an average coverage of 10 to 23 consensus reads per locus. All obtained genotypes are given in Additional file 10.

**Table 3:** Sequencing yield demonstrated high recovery rate for tandem repeats in the assay

		Yield from 3 SMRT cells	
Sequencing yield	Total consensus reads	135,502	
	Unmapped	1696	1.25%
	Within 1000 bases from targets	37,608	27.75%
	Intersecting tandem repeats	32,778	24.19%
	Spanning tandem repeats	29,024	21.42%
	Useful reads (spanning tandem repeats with 20 nt flanks)	26,855	19.82%
Useful reads per target	Average	10.2 - 22.8	
	Median	10–23	
	Maximum	63–166	
Sequenced targets	Total repeats	88.4% - 93.3%	
	‘Functional’ repeats	75.6% - 85.9%	
	‘Non-functional’ repeats	99.0% - 99.6%	

For tandem repeats with moderate GC content (including non-functional repeats) capture and sequencing success reaches 99.0–99.6% ( Additional file 3: Figure S3; Table 3), although for functional targets with high GC content it is ~ 70% lower. We obtained full sequences for almost all GC-poor loci (< 40% GC), while for GC-rich regions (> 70% GC) we observe a decrease in recovery rate to ~ 30% ( Additional file 3: Figure S3) despite the equal probe quadruplication for both groups. Efficiency of a corresponding probe combination is given for each targeted tandem repeat in the Additional file 2 (Sheet 2) together with the influence of the GC content, number of available probe types, total number of the used probes, and full length of a repetitive locus.

### Expansions in large tandem repeats are even detected with partial reads

Because highly expanded alleles could exceed the size of fragments enriched in the libraries, we searched for loci that exclusively yielded reads that did not span the complete repeat. An example of such a large repeat expansion detected by this assay is an intronic repeat in the *CLCN5* gene, represented by 15 copies of 26 bp in the reference genome. We estimated copy numbers for all partial alleles in the sequenced individuals, and the longest ones correspond to at least 24–35 copies (Additional file 10). Thus, the full repeat length of the longest allele is estimated to be more than 900 bp, which is considerably longer than the reference repeat length of 390 bp. This expanded repeat is present in both affected and unaffected individuals in all three families, and since it is located outside the linkage interval in 2 families, the repeat is likely not a causal variant for XLID.

### XLID25 expansion in L020 is potentially linked to the phenotype

Apart from individual repeats, we also included clustered tandem repeats in our analysis. The following strategy was used in each family to narrow down the list of candidate variants. Loci within the linkage intervals, which provided a different unit copy number in the affected versus the unaffected male were then genotyped in other family members by fragment analysis. Moreover, linkage analysis was repeated using these polymorphic repeats as additional segregation markers, allowing to refine the linkage intervals for L020 and L084 families (Table 2; Additional files 11, 12 and 13). Since we expected that mutations in repeat copy number occurred independently in these three XLID families, we checked for unique copy numbers in the patients, which segregated with the phenotype in the family and were absent in the other families. The existence of such variants in the general population was further screened for in a control sampling of up to 100 males. Only copy number variants that were not found in controls were then considered as ID candidate loci.

For the L084 family, 18 tandem repeats were targeted by our assay in the linkage interval, of which 17 are successfully sequenced. Of those, only one intergenic tandem repeat at chrX:145,340,826-145,341,025 (XLID32) exhibits a copy number difference between the affected (78X19) and unaffected (78X28) individuals. However, this allele is also found in control samples and thus considered to be a benign variant.

For the L061 family, we obtained 315 presumably functional loci within the linkage interval with a coverage of at least 5X. Forty five of these repeats were found to be polymorphic within the normal variation range obtained in other XLID families; fragment analysis demonstrated that 3 variants (XLID75, XLID77, XLID79) were false positives; 1 other variant (XLID76) was in the normal size range when compared to additional controls (Table 4); and for 4 repetitive loci (XLID73, XLID74, XLID78, XLID80) the apparent unique copy number detected only in the affected males of the L061 family were also found in the unaffected control population (Table 4). Finally, the tandem repeat XLID72, which displays a shorter polyglutamine tract in the proband compared to his unaffected relative and other families, is located in the first exon of the well characterized *AR* gene. Since the array length of the tandem repeat chrX:66,765,149-66,765,262 is within the normal range, it is considered to be not related to ID.

**Table 4:** Polymorphic tandem repeats in family L061 that were also detected in a control sampling

Chr	Start	End	Origin	Unit length	Copies	Purity	Unit seq.	Annotation	L061 (=37SX) family				Control unaffected males																	
									SN	LJ		Proband	CJ	VHB	LT	DVS	VA	RG	6	8	15	17	20	22	26	49	50	60	61	86
chrX	70,151,351	70,151,390	**XLID73**	2	20	100	GT	Upstream	218	227	**231**	**231**	**231**	229	229	229	**231**	**231**	227	229	229	221	227	225	223					
chrX	74,743,332	74,743,375	**XLID74**	2	22	100	AC	Upstream	227	231	**233**	**233**	227	227	225	229	227	227	227	225	227	227	225	231	227	227	227	225	235	**233**
chrX	84,343,323	84,343,351	**XLID76**	1	29	100	T	NonCoding	356	361	**368**	**368**	361	356	362	360	356	362	362	356	362	356	362	356	356	375	356			
chrX	84,499,126	84,499,197	**XLID78**	3	24	84.7	CGG	FivePrimeUTR	473	463	**463**	**463**							**463**											
chrX	106,184,602	106,184,641	**XLID80**	2	20	100	GA	ThreePrimeUTR	302	300	**310**	**310**	298	290	304	294	298	298	304	304	**310**	304	**310**	312	302					

Proband – affected family member, SN – unaffected male, LJ – carrier of the disease-related haplotype. Allele size is given in base pairs. Alleles found in the individual with ID are in bold: 231, 233, 368, 463 and 310 for the tandem repeats XLID73, XLID74, XLID76, XLID78 and XLID80 respectively

For the L020 family, 44 targeted tandem repeats are present in the linkage region, of which 43 are successfully sequenced. Of these, 5 repeats located in regulatory regions exhibit a copy number difference between the affected (5X20) and unaffected (5X15) family members. However, following fragment analysis, in 4 loci (XLID2, XLID20, XLID22, XLID27) alleles detected in 5X20 and his affected relatives were also detected in 20 control samples (Table 5).

**Table 5:** Repeats in L020 family confirmed potential phenotypical relevance for XLID25 allele upon a control screening

`	Start	End	Name code	Unit length	Copies	Purity	Unit seq.	Annotation	L020 (=5X) family									Control unaffected males													
									5X4		5X7		5X15	5X19	5X17	5X18	Pro-band	Co4	Co5	Co7	Co8	LG	GW	LR	MA	VF	RL	DPS	GR	VR	PB
chrX	44,007,461	44,007,502	**XLID2**	2	21	90.5	GT	ThreePrimeUTR	**354**	364	**354**	364	364	364	**354**	**354**	**354**	**354**	**354**	358	364	**354**	368	362	**354**	**354**	**354**	**354**	364	**354**	356
chrX	45,046,714	45,046,751	**XLID20**	2	19	100	CA	oregannoTFBS	230	**234**	230	**234**	230	230	**234**	**234**	**234**	238	232	228	228	236	230	236	**234**	230	230	232	230	**234**	226
chrX	45,386,687	45,386,738	**XLID22**	2	26	100	CA	Downstream	122	**134**	122	**134**	122	122	**134**	**134**	**134**	138	132	130	**134**	**134**	132	122							
chrX	45,606,270	45,606,355	**XLID25**	2	43	79.1	GT	Downstream	372	**376**	372	**376**	372	372	**376**	**376**	**376**	354	366	372	366	372	366	364	372	374	366	372	372	372	366
chrX	45,709,592	45,709,631	**XLID27**	2	20	100	GT	NonCoding	**415**	421	**415**	421	421	421	**415**	**415**	**415**	**415**	**415**	419	424	**415**	**415**	423							

Proband, 5X17, 5X18 – affected family members, 5X15, 5X19 – unaffected males, 5X4, 5X7 – carriers of the disease-related haplotype. Allele size is given in base pairs. Alleles found in the individual with ID are in bold: 354, 234, 134, 376 and 415 for the tandem repeats XLID2, XLID20, XLID22, XLID25 and XLID27 respectively

**Fig. 2:**
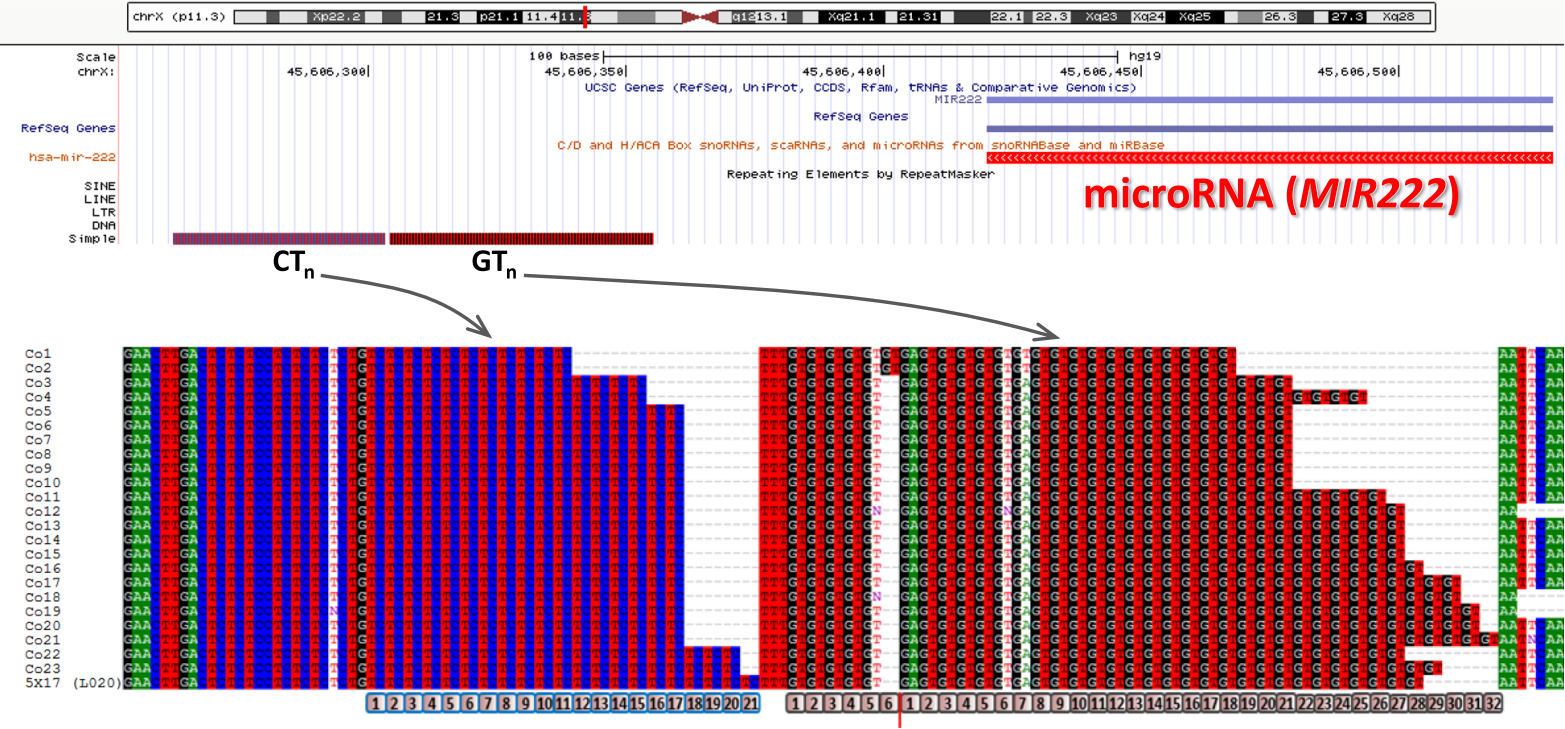
ChrX:45,606,270-45,606,355 tandem repeat (XLID25) is located downstream of *MIR222*. Sanger sequencing revealed the longest (CT)_n_ sub-repeat in L020 XLID patient (5X17, bottom line), compared to 23 unaffected males. Co18 is the unaffected sibling (5X19) from the same L020 family.

The tandem repeat at chrX:45,606,270-45,606,355 (XLID25), which reveals a unique allele in the affected family members segregating with the phenotype, is located 65 bp downstream of the microRNA gene *MIR222*. It is a complex repeat consisting of two consecutive (CT)_n_ and (GT)_n_ sub-repeats separated by three thymidines (Fig. 2). After screening the entire control population of 100 healthy males by fragment analysis only two exhibited the same total length as in the L020 proband. We performed an additional screening by fragment analysis to differentiate (CT)n and (GT)n sub-repeats in those controls (Table S3). For that, we used a nested PCR primer annealing to the boundary that separates the sub-repeats, which gives an approximate estimation of CT copy number. To define the exact length of each individual repeat, amplicons of the 5X17 proband along with that of 23 control males were Sanger sequenced (Fig. 2). The sequenced controls included the samples with the estimated CT copy number equal to or one copy shorter than that of the proband, and the samples with the allele of the same total length as the proband. The latter control samples demonstrated different copy numbers for both (CT)_n_ and (GT)_n_ sub-repeats (17 CT and 37 GT copies or 20 CT and 34 GT copies), compared to the proband who exhibited an allele with the highest CT copy number (21), while the GT unit number (33) was in the normal range of 24–37 copies, observed in the controls. Notably, none of the sequenced samples revealed the (CT)_n_ sub-repeat longer than 20 copies.

### Upstream *MIR222* gene reveals decreased expression, and its targets *FMR1*, *NEFH* are up-regulated in the proband

**Fig. 3:**
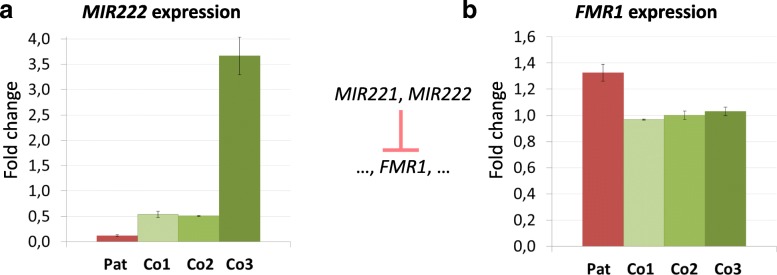
The expression level of *MIR222* (**a**) is decreased while one of its targets, *FMR1* (**b**)*,* is up-regulated in the patient (‘Pat’, red) opposed to 3 controls (‘Co1’, ‘Co2’, ‘Co3’, shades of green). Error bars show standard deviation of the normalized expression in 2 and 3 experiments, respectively. ‘Co1’, ‘Co2’, ‘Co3’ samples correspond to ‘Co14’ (17 CT, 32GT), ‘Co15’ (17 CT, 32GT), ‘Co17’ (17 CT, 35GT) males in the sequence alignment in Fig. 2

To investigate the possible effect of the XLID25 CT-sub-repeat expansion on *MIR222* expression, we performed a miRNA specific TaqMan assay on the enriched small-RNA samples extracted from EBV-PBL cell lines of the proband and three male controls. As no cell lines were available for the other family members, their *MIR222* expression has not been tested. The results show that *MIR222* expression is decreased at least 5-fold in the patient (Fig. 3a) compared to the control samples that correspond to ‘Co14’ (genotype 17 CT, 32GT), ‘Co15’ (17 CT, 32GT) and ‘Co17’ (17 CT, 35GT) males in the sequence alignment in Fig. 2. Additionally, we tested the expression of one of the *MIR222* downstream targets, *FMR1,* known to be involved in intellectual disability. Interestingly, the *FMR1* mRNA levels are elevated by 30% in the XLID patient compared to the 3 controls, who show highly similar levels (Fig. 3b).

**Fig. 4:**
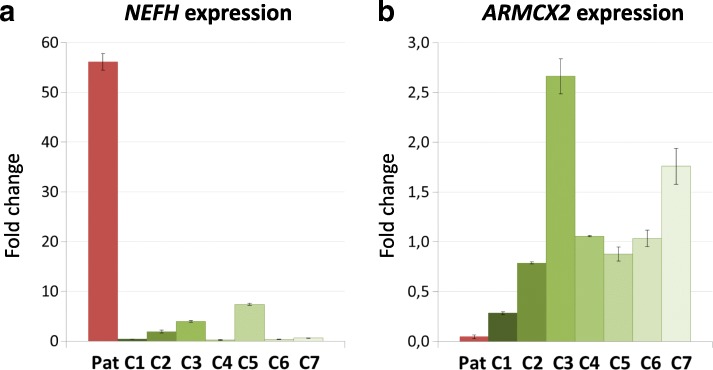
Up-regulated expression of *NEFH* (**a**) and down-regulation of *ARMCX2* mRNA (**b**) in the affected proband (‘Pat’, red) and 7 controls (‘C1–7’, shades of green). Error bars show standard deviation of the normalized expression in 3 experiments

To reveal additional deregulated genes that might be affected by the altered *MIR222* expression, we performed RNA sequencing again on RNA extracted from EBV-PBLs of the patient and 3 controls, same as in the previous experiment. We obtained 46 loci that are significantly and consistently up- or down-regulated in the patient, however none of them is within the linkage interval of the family. Based on Ingenuity Pathway Analysis most of these genes are involved in anatomical structure morphogenesis, cellular component movement, locomotion and localization of the cell. R ratio ≥ 0.9 for expression deregulation in patient is observed in 35 genes, of which 31 are known to be expressed in brain. Of these, 21 genes (68%) are predicted targets of *MIR222*, while it is expected to regulate half as many (30%) in an entire pool of the brain-expressed genes. Nine genes were selected to confirm the RNAseq data by qPCR. Upon increasing the number of controls to 7, the altered expression in the patient remained apparent in 2 of the 9 selected genes: *ARMCX2* and *NEFH* (Fig. 4), which are predicted targets of *MIR222* (microRNA.org). Notably, the *NEFH* gene encoding the heavy neurofilament protein reveals a 52-fold increased expression level in the XLID patient in relation to the 7 controls. As for the genes within the linkage interval other than *MIR222*, for 7 of them (*MAOA, DUSP21, PPP1R2P9, MAOB, NDP, EFHC2* and *MIR221*) we did not obtain any RNA sequencing data while other 8 (*KDM6A, ZNF673, FUNDC1, CXorf36, ZNF674, KRBOX4, ZNF674-AS1* and *CHST7*) reveal expression differences that are not statistically significant (*p*-values 0.29–0.99).

## Discussion

Despite multiple large-scale array-CGH and exome and genome sequencing analyses, the genetic variation underlying X-linked intellectual disabilities remains elusive for a large number of families. We hypothesize that tandem repeat expansions have escaped detection mainly due to short-read sequencing technologies. Here, we developed an extensive screen for X linked tandem repeat expansions using a long read MPS approach. We captured and sequenced more than 1800 tandem repeats in three families with idiopathic XLID and demonstrate the feasibility of single molecule sequencing to accurately detect and size tandem repeats and tandem repeat variability. Moreover, in one family a tandem repeat expansion segregating with ID seems to affect the expression of the nearby *MIR222* gene, previously implicated in ID [42] and affecting expression levels of genes associated with ID [43–46].

Multiple studies have been reported on large-scale analysis of tandem repeat polymorphism in several species, with various enrichment strategies, and utilizing different massively-parallel sequencing technologies. Information on thousands of repeats was previously retrieved from whole-genome or targeted sequencing data for 8 individual genomes [47], 550 and later 1009 individuals from the 1000 Genomes Project [48, 49], or 34 human gastric cancer cell lines and tissue samples [50]. An extensive characterization of > 390 thousand loci in more than 150 *Drosophila* strains by Fondon et al. [51] provided an important framework for further association studies in this species. However, Illumina read length limits target loci to microsatellites shorter than ~ 90 bp or less. On the contrary, the use of the 454 platform, generating longer reads, allowed to discover hundreds to thousands of new microsatellites in 14 species among animal, plant and fungi taxa [52], or selectively target human tandem repeats with a broader range of characteristics [30] with a total length up to ~ 300 bp. More recently, long read single molecule sequencing has been introduced to study tandem repeats, though limited to only a few loci so far [21–23]. Here, we combined the benefits of broad-range targeted sequencing with the large read size, which permitted analysis of virtually all coding and regulatory repeats on the X chromosome. Overall, 83% of all likely functional repeats could be targeted with unique probes, and on average more than 90% of targets were captured and sequenced.

Capture-based enrichment has previously been applied in studies of tandem repeat variation. The first approach [52] used 8 spanning probes designed to bind all microsatellites in the genome with probe-matching motifs. Later studies focused on a more selective and specific capture approach using probes complementary to the unique flanking regions of such repeats, which boosted the capture efficiency. Guilmatre et al. [47] utilized solely flanking probes and increased the on-target sequences to 39%. The design of longer flanking probes, as well as the addition of spanning and special double-flank probes that we previously described even raised the capture efficiency to 62% [30]. Our current probe design, based on the latter approach, however, resulted in a lower on-target rate of 28%. This apparent capture efficiency drop is likely due to a more than 5-time decrease in the number of targets compared to Duitama et al. [30]. According to the manufacturer (personal communication), this is a frequently observed effect of excess baits that are forced to bind aspecifically when there are not enough target molecules for a proper hybridization. Hence, it was suggested to test half of the recommended bait amount for the future hybridizations. Another difference in the probe design that probably has a minor effect is the ratio of the probes with extreme GC content. A higher percentage of probes with low GC content is likely to hybridize non-specifically to AT-rich Alu elements that are abundantly present in the genome, thereby increasing the off-target count. However, despite the large off-target rate our assay demonstrated high recovery rate of the targeted regions. It is clear though that optimizing the baits stoichiometry and further improvements to the probe design have the potential to boost the capture efficiency even more.

Interestingly, tandem repeat expansions can be detected even if the expansion exceeds the targeted fragment length. As an example, we detected expansion of more than 900 bp for an intronic tandem repeat in *CLCN5* that is only 390 bp long in the reference genome. This expansion would not be detected with short-read technologies. We found such elongated alleles in all six sequenced individuals suggesting either a significant length variation in the population, or a local misassembly and a hidden gap in the reference genome. If so, this would not be surprising considering that multiple studies find repeat arrays in remaining gaps of genome assemblies [53–55].

A series of tandem repeats were shown to be variable in length between affected and unaffected relatives. However, all but one did not seem to be associated with ID as they were also found in healthy controls or were in disagreement with the clinical description. In one of the families (L020), a single repeat (‘XLID25’) consisting of two adjacent tandem repeat stretches revealed a unique expanded allele of 21 copies in the CT sub-repeat in the proband, whereas all other genotyped healthy individuals of the family as well as controls had unit numbers in the range of 11–20 copies.

CT-repeats (or GA-repeats in complement), also known as GAGA-elements, act as chromatin remodelling mediators by disrupting and displacing pre-assembled nucleosomes [56–58]. Emamalizadeh and colleagues [59] suggested that the (GA)_11_ repeat length in the promoter of *RIT2* is crucial for obtaining the correct dosage of RIT2, important in regulating the neuronal function. A shorter allele (GA)_5_ in homozygous state has been detected only in a proband with schizophrenia thus linking it to the disease state. Similarly, length variation of (GA)_n_ tandem repeats influences embryonic development and facilitates evolutionary adaptation by regulating *MECOM* and *GABRA3* expression [60]. It has been reported that GAGA-binding protein in humans specifically binds to the elements of 8 GA-units [61], which explains why precise copy number ranges are extremely important in regulatory sites. GA-dinucleotides may also affect gene expression when located downstream of that gene [62], which is in agreement with our data.

Only 65 bp upstream of XLID25 a highly conserved microRNA gene, *MIR222,* is located. *MIR222* is mainly expressed in telencephalon with a conserved pattern of expression in larval and adult brain in zebrafish [63]. The *MIR221/222* cluster is known to play an important role in coordination of cell proliferation [64]. They were shown to regulate terminal differentiation of neurons in porcine cortex and cerebellum [65]. Moreover, *MIR222* was demonstrated to regulate timing of neural development by blocking preliminary generation of bipolar neurons in *Xenopus* [66]. It has been suggested that *MIR222,* with or without *MIR221,* is a plausible candidate to cause intellectual disability associated with X-linked retinal dystrophy in the Xp11.3 deletion syndrome [42]. Interestingly, in a study by Chen et al. [67], screening of 13 brain-expressed miRNAs in 464 patients with X-linked intellectual disability revealed only 4 mutations, of which 2 segregated with the phenotype, and both were found in *MIR222*. One of these mutations was located near the Drosha ribonuclease cleavage site and therefore, could potentially affect mature miRNA formation. On the other hand, the high conservation of the brain-expressed *MIR222* suggests an important function in this tissue. We demonstrated that in the proband of the L020 family expression levels of *MIR222* were decreased. In consonance with our study, *MIR222* and *MIR221* levels were previously found to be down-regulated in hippocampal tissue of patients with a neurological disease – mesial temporal lobe epilepsy and hippocampal sclerosis [68]. All these studies point to a crucial role of *MIR222* for proper brain functioning. Decreased levels seem to disturb yet enigmatic neuronal processes.

We also tested the expression of one of the *MIR222* targets namely *FMR1*. FMRP is an mRNA binding protein that has multiple functions in post-transcription gene regulation including mRNA stability, mRNA transport and localization, translation control, and pre-mRNA alternative splicing [69] with the latter being more prevalent in brain compared to other tissues [70]. *FMR1* is usually the primary gene to test in the newly diagnosed ID patients [4]. Large tandem repeat expansions cause silencing of this gene that leads to intellectual disability with more severe forms in males. Contrary to this most common mechanism, we demonstrated a significantly increased expression of *FMR1* in the affected male of L020. This controversy may suggest that precise concentration of FMRP in the brain is required for its proper function, and any dysregulation disrupting the balance can cause a disease. Our data is in line with the fact that the gene was shown to cause abnormal behavior when overexpressed in mice, a specific high-anxiety phenotype that is different from *FMR1* knock-out mice [71]. Elevated *FMR1* expression levels were also suggested to be causal in 5 ID patients carrying a duplication that harbors the *FMR1* gene, amongst several others [43–46]. However, only one study [44] looked into mRNA expression in blood of a proband, which was within the normal range. Either the presence of two copies in males is not sufficient to cause overexpression or the expression levels in brain are different from those in blood cells leading to tissue-specific consequences.

In order to detect additional genes that could be regulated by *MIR222*, we performed total RNA sequencing and detected a second deregulated target of *MIR222* namely *NEFH*. Its product is a component of neural cytoskeleton important for neuronal maintenance and plasticity, neurite outgrowth, axonal caliber and transport [72]. Interestingly, in the L020 proband this gene was 50-fold up-regulated. As shown by Collard et al. [73] overexpression of human *NEFH* in mice causes defects in axonal transport, which eventually leads to neuron degeneration. Notably, NEFH protein was described 1.5-fold up-regulated in children with cortical dysplasia with epilepsy [74].

All these findings provide indirect evidence that the unique tandem repeat variant of 21 copies of the CT/GA repeat is a strong candidate for the ID phenotype in the L020 family. The expanded repeat might cause decreased expression of the nearby *MIR222* microRNA resulting in a decreased breakdown of neuronal target genes including *FMR1* and *NEFH*.

To confirm a causal link between the detected tandem repeat variant and the XLID phenotype, further studies are required. Future screening of affected cohorts and healthy population for XLID25 expansions, microRNA *MIR222* mutations*,* expression profiles of *MIR222* might reveal more cases and statistical significant associations. In addition, cellular experiments will assess the impact of the repeat size on the *MIR222* expression in the same patient cell line. Potentially, CRISPR/Cas9-induced double-strand break within the tandem repeat will lead to the reparation-induced repeat instability [75, 76]. This should allow to generate cell lines with a common genetic background and only different by the repeat copy number which would provide a reliable functional model. Finally, a knock-out of the microRNA in a mouse model would demonstrate its role in the XLID development.

To our knowledge, this is the first study capturing and single molecule sequencing targeted genomic loci. This strategy can be applied to other targets as well as to repeats elsewhere in the genome. Though this particular probe design is only applicable to the X-linked disorders, our tandem repeats screening approach is expandable to other chromosomes. With time, improved genome annotation might require an update of the list of tandem repeats that are potentially relevant for a disease development.

Whereas our method allows the detection of a majority of repeat expansions, it might fail on longer repeats. In this study, we targeted repeats up to 500 bp. This enabled sequencing over the repeat multiple times, generating proper consensus lengths. To accurately measure repeat lengths, the polymerase reads must be at least six times the size of the insert. With a mean polymerase read length of 15 kb, the repeat sizes should have a maximum length of 2–2.5 kb. Nevertheless, with ever expanding longer reads, the repeats sizes that can be measured, will also be expanded. However, such long repeats constitute only a small portion of all repeats in the genome. A second limitation is that the capture method has a reduced performance on GC-rich tandem repeats or fragments. Although the single molecule sequencing has no problem passing GC-rich repeats, those sequences show reduced capture efficiency.

## Conclusions

Our findings provide indirect evidence that the unique tandem repeat variant of 21 copies of the CT/GA repeat is a strong candidate for the ID phenotype in one of the studied families with X-linked ID. The expanded repeat might cause decreased expression of the nearby *MIR222* microRNA resulting in a decreased breakdown of neuronal target genes including *FMR1* and *NEFH*. Present work is the first large-scale study of targeted sequencing of tandem repeats as a means to improve diagnosis of an inherited disease. Future application of the described assay in a large number of cases will allow to evaluate the general contribution of tandem repeat instability to XLID. Next to XLID, our design may be used to study other X chromosome related diseases too. Moreover, this approach is not restricted to the X chromosome, but is applicable to screen for tandem repeats on other chromosomes as well.

## Additional files

Additional file 1: Custom script searching for unique capture probes that are closest to the target within a specified distance. (SH 13 kb)

Additional file 2: SureSelect capture probe design and information on the performance of the different probe combinations per target. (XLSX 1353 kb)

Additional file 3: **Tables S1–S2**, supplemental **Figures S1–S3**, sample tables of the Additional files 2, 9 and 10. (PDF 810 kb)

Additional file 4: Custom script removing adaptor sequences from the sequencing reads. (PY 1 kb)

Additional file 5: Custom script for estimating the number of reads spanning a tandem repeat together with its 20 nt-long flanking regions, considering soft-clipped reads with several partial alignments to the flanks. (SH 2 kb)

Additional file 6: Supplementary script called by the countTargetSpanning.sh script (Additional file 5). (PY 2 kb)

Additional file 7: Custom script filtering the reads that were mapped within a specified distance from the targeted region, and creating a separate input file for the TSSV tool. (PY 11 kb)

Additional file 8: Custom script processing the output of the TSSV tool and estimating the genotype of a tandem repeat. (PY 30 kb)

Additional file 9: List of primers used in this study. (XLSX 21 kb)

Additional file 10: Genotypes obtained from the sequencing data of the targeted tandem repeats in three families with X-linked intellectual disability. (XLSX 2711 kb)

Additional file 11: Output data from the linkage analysis in the L020 family with X-linked intellectual disability. (LOG 4 kb)

Additional file 12: Output data from the linkage analysis in the L061 family with X-linked intellectual disability. (LOG 13992 kb)

Additional file 13: Output data from the linkage analysis in the L084 family with X-linked intellectual disability. (LOG 4 kb)

## Abbreviations

aCGH: array-based comparative genomic hybridization
EBV-PBL: Peripheral blood lymphocytes transformed with Epstein Barr Virus
GC: Guanine-cytosine
ID: Intellectual disability
LOD: Logarithm (base 10) of odds
miRNA: microRNA
MPS: Massively-parallel sequencing
SMRT: Single molecule real-time
XLID: X chromosome-linked intellectual disability

## References

[1] Ropers HH (2008). Genetics of intellectual disability. Curr Opin Genet Dev.

[2] Gécz J, Shoubridge C, Corbett M (2009). The genetic landscape of intellectual disability arising from chromosome X. Trends Genet.

[3] Kleefstra T, Yntema H, Oudakker A, Banning M, Kalscheuer V, Chelly J, Moraine C, Ropers H, Fryns J, Janssen I (2004). Zinc finger 81 (*ZNF81*) mutations associated with X-linked mental retardation. J Med Genet.

[4] de Brouwer AP, Yntema HG, Kleefstra T, Lugtenberg D, Oudakker AR, de Vries BB, van Bokhoven H, Van Esch H, Frints SG, Froyen G (2007). Mutation frequencies of X-linked mental retardation genes in families from the EuroMRX consortium. Hum Mutat.

[5] Kousoulidou L, Parkel S, Zilina O, Palta P, Puusepp H, Remm M, Turner G, Boyle J, Van Bokhoven H, de Brouwer A (2007). Screening of 20 patients with X-linked mental retardation using chromosome X-specific array-MAPH. Eur J Med Genet.

[6] Jensen LR, Chen W, Moser B, Lipkowitz B, Schroeder C, Musante L, Tzschach A, Kalscheuer VM, Meloni I, Raynaud M (2011). Hybridisation-based resequencing of 17 X-linked intellectual disability genes in 135 patients reveals novel mutations in *ATRX*, *SLC6A8* and *PQBP1*. Eur J Hum Genet.

[7] Van Esch H, Bauters M, Ignatius J, Jansen M, Raynaud M, Hollanders K, Lugtenberg D, Bienvenu T, Jensen LR, Gecz J (2005). Duplication of the *MECP2* region is a frequent cause of severe mental retardation and progressive neurological symptoms in males. Am J Hum Genet.

[8] Froyen G, Van Esch H, Bauters M, Hollanders K, Frints SG, Vermeesch JR, Devriendt K, Fryns JP, Marynen P (2007). Detection of genomic copy number changes in patients with idiopathic mental retardation by high-resolution X-array-CGH: important role for increased gene dosage of XLMR genes. Hum Mutat.

[9] Bashiardes S, Kousoulidou L, Van Bokhoven H, Ropers HH, Chelly J, Moraine C, de Brouwer AP, Van Esch H, Froyen G, Patsalis PC (2009). A new chromosome x exon-specific microarray platform for screening of patients with X-linked disorders. J Mol Diagn.

[10] Isrie M, Froyen G, Devriendt K, de Ravel T, Fryns JP, Vermeesch JR, Van Esch H (2012). Sporadic male patients with intellectual disability: contribution of X-chromosome copy number variants. Eur J Med Genet..

[11] Hu H, Wrogemann K, Kalscheuer V, Tzschach A, Richard H, Haas SA, Menzel C, Bienek M, Froyen G, Raynaud M (2009). Mutation screening in 86 known X-linked mental retardation genes by droplet-based multiplex PCR and massive parallel sequencing. HUGO J.

[12] de Ligt J, Willemsen MH, van Bon BWM, Kleefstra T, Yntema HG, Kroes T, Vulto-van Silfhout AT, Koolen DA, de Vries P, Gilissen C (2012). Diagnostic exome sequencing in persons with severe intellectual disability. N Engl J Med.

[13] Gilissen C, Hehir-Kwa JY, Thung DT, van de Vorst M, van Bon BW, Willemsen MH, Kwint M, Janssen IM, Hoischen A, Schenck A (2014). Genome sequencing identifies major causes of severe intellectual disability. Nature.

[14] Hu H, Haas SA, Chelly J, Van Esch H, Raynaud M, de Brouwer APM, Weinert S, Froyen G, Frints SGM, Laumonnier F (2016). X-exome sequencing of 405 unresolved families identifies seven novel intellectual disability genes. Mol Psychiatry.

[15] Tarpey PS, Smith R, Pleasance E, Whibley A, Edkins S, Hardy C, O'Meara S, Latimer C, Dicks E, Menzies A (2009). A systematic, large-scale resequencing screen of X-chromosome coding exons in mental retardation. Nat Genet.

[16] Huang L, Jolly LA, Willis-Owen S, Gardner A, Kumar R, Douglas E, Shoubridge C, Wieczorek D, Tzschach A, Cohen M (2012). A Noncoding, regulatory mutation implicates *HCFC1* in nonsyndromic intellectual disability. Am J Hum Genet.

[17] Legendre M, Pochet N, Pak T, Verstrepen KJ (2007). Sequence-based estimation of minisatellite and microsatellite repeat variability. Genome Res.

[18] Vinces MD, Legendre M, Caldara M, Hagihara M, Verstrepen KJ (2009). Unstable tandem repeats in promoters confer transcriptional evolvability. Science.

[19] Gemayel R, Vinces MD, Legendre M, Verstrepen KJ (2010). Variable tandem repeats accelerate evolution of coding and regulatory sequences. Annu Rev Genet.

[20] Bilgin Sonay T, Carvalho T, Robinson MD, Greminger MP, Krützen M, Comas D, Highnam G, Mittelman D, Sharp A, Marques-Bonet T (2015). Tandem repeat variation in human and great ape populations and its impact on gene expression divergence. Genome Res.

[21] Guo X, Zheng S, Dang H, Pace RG, Stonebraker JR, Jones CD, Boellmann F, Yuan G, Haridass P, Fedrigo O (2014). Genome reference and Sequence variation in the large repetitive central exon of human *MUC5AC*. Am J Respir Cell Mol Biol.

[22] McFarland KN, Liu J, Landrian I, Godiska R, Shanker S, Yu F, Farmerie WG, Ashizawa T (2015). SMRT sequencing of long tandem nucleotide repeats in SCA10 reveals unique insight of repeat expansion structure. PLoS One.

[23] Ardui S, Race V, Zablotskaya A, Hestand MS, Van Esch H, Devriendt K, Matthijs G, Vermeesch JR (2017). Detecting AGG interruptions in male and female *FMR1* Premutation carriers by single-molecule sequencing. Hum Mutat.

[24] UCSC Sequence and Annotation Downloads, Feb. 2009 Assembly of the human genome. http://hgdownload.soe.ucsc.edu/goldenPath/hg19/chromosomes/chrX.fa.gz. Accessed 10 Sept 2013.

[25] Rice P, Longden I, Bleasby A (2000). EMBOSS: the European molecular biology open software suite. Trends Genet.

[26] UCSC Sequence and Annotation Downloads, Feb. 2009 Assembly of the human genome. http://hgdownload.soe.ucsc.edu/goldenPath/hg19/database/microsat.txt.gz. Accessed 10 Sept 2013.

[27] Short Tandem Repeat DNA Internet DataBase. http://www.cstl.nist.gov/biotech/strbase/. Accessed 10 Sept 2013.

[28] Orr HT, Zoghbi HY (2007). Trinucleotide repeat disorders. Annu Rev Neurosci.

[29] UCSC Sequence and Annotation Downloads, Feb. 2009 Assembly of the human genome. http://hgdownload.soe.ucsc.edu/goldenPath/hg19/database/simpleRepeat.txt.gz. Accessed 10 Sept 2013.

[30] Duitama J, Zablotskaya A, Gemayel R, Jansen A, Belet S, Vermeesch JR, Verstrepen KJ, Froyen G (2014). Large-scale analysis of tandem repeat variability in the human genome. Nucleic Acids Res.

[31] Greenwood Genetic Center. https://www.ggc.org/xlid-genetic-research. Accessed 20 Apr 2014.

[32] Langmead B, Trapnell C, Pop M, Salzberg SL (2009). Ultrafast and memory-efficient alignment of short DNA sequences to the human genome. Genome Biol.

[33] Claes S, Volcke P, Devriendt K, Holvoet M, Raeymaekers P, Cassiman JJ, Fryns JP. Regional localization of a gene for nonspecific XLMR to Xp11.3-p11.23 (MRX51) and tentative localization of an MRX gene to Xq23-q26.1. Am J Med Genet. 1999, 85:283–7. 10398244

[34] Li H, Durbin R (2010). Fast and accurate long-read alignment with burrows-wheeler transform. Bioinformatics.

[35] Picard. http://broadinstitute.github.io/picard. Accessed 14 Aug 2014.

[36] Quinlan AR, Hall IM (2010). BEDTools: a flexible suite of utilities for comparing genomic features. Bioinformatics.

[37] Anvar SY, van der Gaag KJ, van der Heijden JW, Veltrop MH, Vossen RH, de Leeuw RH, Breukel C, Buermans HP, Verbeek JS, de Knijff P (2014). TSSV: a tool for characterization of complex allelic variants in pure and mixed genomes. Bioinformatics.

[38] Rozen S, Skaletsky H, Krawetz S, Misener S (2000). Primer3 on the WWW for general users and for biologist programmers. Bioinformatics methods and protocols: methods in molecular biology.

[39] Trapnell C, Pachter L, Salzberg SL (2009). TopHat: discovering splice junctions with RNA-Seq. Bioinformatics.

[40] Li H, Handsaker B, Wysoker A, Fennell T, Ruan J, Homer N, Marth G, Abecasis G, Durbin R (2009). 1000 genome project data processing subgroup. The Sequence alignment/map (SAM) format and SAMtools. Bioinformatics.

[41] Trapnell C, Williams BA, Pertea G, Mortazavi A, Kwan G, van Baren MJ, Salzberg SL, Wold BJ, Pachter L (2010). Transcript assembly and quantification by RNA-Seq reveals unannotated transcripts and isoform switching during cell differentiation. Nat Biotechnol.

[42] Delphin N, Hanein S, Taie LF, Zanlonghi X, Bonneau D, Moisan J-P, Boyle C, Nitschke P, Pruvost S, Bonnefont J-P (2012). Intellectual disability associated with retinal dystrophy in the Xp11.3 deletion syndrome: *ZNF674* on trial. Guilty or innocent?. Eur J Hum Genet.

[43] Rio M, Malan V, Boissel S, Toutain A, Royer G, Gobin S, Morichon-Delvallez N, Turleau C, Bonnefont J-P (2010). familial interstitial Xq27.3q28 duplication encompassing the *FMR1* gene but not the *MECP2* gene causes a new syndromic mental retardation condition. Eur J Hum Genet.

[44] Vengoechea J, Parikh AS, Zhang S, Tassone F (2012). *De novo* microduplication of the *FMR1* gene in a patient with developmental delay, epilepsy and hyperactivity. Eur J Hum Genet.

[45] Nagamani SCS, Erez A, Probst FJ, Bader P, Evans P, Baker LA, Fang P, Bertin T, Hixson P, Stankiewicz P (2012). Small genomic rearrangements involving *FMR1* support the importance of its gene dosage for normal neurocognitive function. Neurogenetics.

[46] Hickey SE, Walters-Sen L, Mosher TM, Pfau RB, Pyatt R, Snyder PJ, Sotos JF, Prior TW (2013). Duplication of the Xq27.3–q28 region, including the *FMR1* gene, in an X-linked hypogonadism, gynecomastia, intellectual disability, short stature, and obesity syndrome. Am J Med Genet Part A.

[47] Guilmatre A, Highnam G, Borel C, Mittelman D, Sharp AJ (2013). Rapid multiplexed genotyping of simple tandem repeats using capture and high-throughput sequencing. Hum Mutat.

[48] McIver LJ, McCormick JF, Martin A, Fondon JW, Garner HR (2013). Population-scale analysis of human microsatellites reveals novel sources of exonic variation. Gene.

[49] Willems T, Gymrek M, Highnam G (2014). The 1000 genomes project consortium, Mittelman D, Erlich Y. the landscape of human STR variation. Genome Res.

[50] Yoon K, Lee S, Han TS, Moon SY, Yun SM, Kong SH, Jho S, Choe J, Yu J, Lee HJ (2013). Comprehensive genome- and transcriptome-wide analyses of mutations associated with microsatellite instability in Korean gastric cancers. Genome Res.

[51] Fondon JW, Martin A, Richards S, Gibbs RA, Mittelman D (2012). Analysis of microsatellite variation in *Drosophila melanogaster* with population-scale genome sequencing. PLoS One.

[52] Malausa T, Gilles A, Meglecz E, Blanquart H, Duthoy S, Costedoat C, Dubut V, Pech N, Castagnone-Sereno P, Delye C (2011). High-throughput microsatellite isolation through 454 GS-FLX titanium pyrosequencing of enriched DNA libraries. Mol Ecol Resour.

[53] Altemose N, Miga KH, Maggioni M, Willard HF (2014). Genomic characterization of large heterochromatic gaps in the human genome assembly. PLoS Comput Biol.

[54] Chaisson MJP, Huddleston J, Dennis MY, Sudmant PH, Malig M, Hormozdiari F, Antonacci F, Surti U, Sandstrom R, Boitano M (2015). Resolving the complexity of the human genome using single-molecule sequencing. Nature.

[55] Miga KH (2015). Completing the human genome: the progress and challenge of satellite DNA assembly. Chromosom Res.

[56] Granok H, Leibovitch BA, Shaffer CD, Elgin SCR (1995). Ga-ga over GAGA factor. Curr Biol.

[57] Lehmann M (2004). Anything else but GAGA: a nonhistone protein complex reshapes chromatin structure. Trends Genet.

[58] Tsai S-Y, Chang Y-L, Swamy KBS, Chiang R-L, Huang D-H (2016). GAGA factor, a positive regulator of global gene expression, modulates transcriptional pausing and organization of upstream nucleosomes. Epigenetics Chromatin.

[59] Emamalizadeh B, Movafagh A, Darvish H, Kazeminasab S, Andarva M, Namdar-Aligoodarzi P, Ohadi M (2017). The human *RIT2* core promoter short tandem repeat predominant allele is species-specific in length: a selective advantage for human evolution?. Mol Gen Genomics.

[60] Valipour E, Kowsari A, Bayat H, Banan M, Kazeminasab S, Mohammadparast S, Ohadi M (2013). Polymorphic core promoter GA-repeats alter gene expression of the early embryonic developmental genes. Gene.

[61] Berger N, Dubreucq B (2012). Evolution goes GAGA: GAGA binding proteins across kingdoms. Biochim Biophys Acta Gene Regul Mech.

[62] Nakayama T, Nishioka K, Dong Y-X, Shimojima T, Hirose S. *Drosophila* GAGA Factor directs histone H3.3 replacement that prevents the heterochromatin spreading. Genes Dev 2007;21(5):552–561. 10.1101/gad.1503407 PMC1820897 17344416

[63] Kapsimali M, Kloosterman WP, de Bruijn E, Rosa F, Plasterk RH, Wilson SW (2007). MicroRNAs show a wide diversity of expression profiles in the developing and mature central nervous system. Genome Biol.

[64] Medina R, Zaidi SK, Liu C-G, Stein JL, van Wijnen AJ, Croce CM, Stein GS (2008). MicroRNAs 221 and 222 bypass quiescence and compromise cell survival. Cancer Res.

[65] Podolska A, Kaczkowski B, Kamp Busk P, Sokilde R, Litman T, Fredholm M, Cirera S (2011). MicroRNA expression profiling of the porcine developing brain. PLoS One.

[66] Decembrini S, Bressan D, Vignali R, Pitto L, Mariotti S, Rainaldi G, Wang X, Evangelista M, Barsacchi G, Cremisi F (2009). MicroRNAs couple cell fate and developmental timing in retina. Proc Natl Acad Sci U S A.

[67] Chen W, Jensen LR, Gecz J, Fryns J-P, Moraine C, de Brouwer A, Chelly J, Moser B, Ropers HH, Kuss AW (2007). Mutation screening of brain-expressed X-chromosomal miRNA genes in 464 patients with nonsyndromic X-linked mental retardation. Eur J Hum Genet.

[68] Kan AA, van Erp S, Derijck AAHA, de Wit M, Hessel EVS, O’Duibhir E, de Jager W, Van Rijen PC, Gosselaar PH, de Graan PNE (2012). Genome-wide microRNA profiling of human temporal lobe epilepsy identifies modulators of the immune response. Cell Mol Life Sci.

[69] Zhoua L-T, Yea S-H, Yanga H-X, Zhoua Y-T, Zhaoa Q-H, Suna W-W, Gaoa M-M, Yia Y-H, Longa Y-S (2017). A novel role of fragile X mental retardation protein in pre-mRNA alternative splicing through RNA-binding protein. Neuroscience.

[70] Raj B, Blencowe BJ (2015). Alternative splicing in the mammalian nervous system: recent insights into mechanisms and functional roles. Neuron.

[71] Peier AM, McIlwain KL, Kenneson A, Warren ST, Paylor R, Nelson DL (2000). (over)correction of *FMR1* deficiency with *YAC* transgenics: behavioral and physical features. Hum Mol Genet.

[72] Al-Chalabi A, Miller CCJ (2003). Neurofilaments and neurological disease. BioEssays.

[73] Collard J-F, Cote F, Julien J-P (1995). Defective axonal transport in a transgenic mouse model of amyotrophic lateral sclerosis. Nature.

[74] Qin L, Liu X, Liu S, Liu Y, Yang Y, Yang H, Chen Y, Chen L (2017). Differentially expressed proteins underlying childhood cortical dysplasia with epilepsy identified by iTRAQ proteomic profiling. PLoS One.

[75] Lv Q, Lai L, Yuan L, Song Y, Sui T, Li Z (2016). Tandem repeat knockout utilizing the CRISPR/Cas9 system in human cells. Gene.

[76] Van Agtmaal EL, André LM, Willemse M (2017). CRISPR/Cas9-induced (CTG·CAG)n repeat instability in the myotonic dystrophy type 1 locus: implications for therapeutic genome editing. Mol Ther.

