# Supplementary material for: Mapping the landscape of tandem repeat variability by targeted long read single molecule sequencing in familial X-linked intellectual disability
Source: BMC Med Genomics. 2018 Dec 19;11:123. doi: 10.1186/s12920-018-0446-7 (PMC6299999; doi:10.1186/s12920-018-0446-7)
Supplement: Supplementary file 3 — Tables S1–S2, supplemental Figures S1–S3, sample tables of the Additional files 2, 9 and 10. (PDF 810 kb) [file 12920_2018_446_MOESM3_ESM.pdf]

Mapping the landscape of tandem repeat variability by targeted long read single molecule sequencing in familial X-linked intellectual disability

Alena Zablotskaya, Hilde Van Esch, Kevin J. Verstrepen, Guy Froyen, Joris R. Vermeesch

Corresponding author: joris.vermeesch@kuleuven.be; Laboratory for Cytogenetics and Genome Research, Center for Human Genetics, University Hospitals Leuven; Department of Human Genetics, KU Leuven, O&N I Herestraat 49 - box 606, 3000 Leuven, Belgium

SUPPLEMENTAL MATERIALS

1. Table S1 p. 2

2. Figure S1 p. 3

3. Additional file 2 (sample table) p. 4

4. Figure S2 p. 5

5. Additional file 9 (sample table) p. 6

6. Table S2 p. 7

7. Figure S3 p. 8

8. Additional file 10 (sample table) p. 9

9. Table S3 p. 10

Table S1 Success of default and modified capture probe design

#	Selection groups	N	Original Probe Design					Probe Design with moved positions (up to 200 nt)				
			>=1 probe	>=2 probes	>=3 probes	4 probes	Untargeted	>=1 probe	>=2 probes	>=3 probes	4 probes	Untargeted
	**Coding repeats only**	**368**	**261**	**236**	**214**	**158**	**92**	**+ 25**	**+ 15**	**+ 2**	**+ 0**	**- 25**
**1**		**353**	**261**	**236**	**214**	**158**	**92**	**+ 25**	**+ 15**	**+ 2**	**+ 0**	**- 25**
1b		9	Excluded from probe design									
1c		6	Excluded from probe design									
	**Regulatory repeats only (no intergenic/intronic, no coding repeats)**	**181**	**112**	**92**	**62**	**12**	**62**	**+ 8**	**+ 6**	**+ 1**	**+ 0**	**- 8**
**2**		**174**	**112**	**92**	**62**	**12**	**62**	**+ 8**	**+ 6**	**+ 1**	**+ 0**	**- 8**
2b		5	Excluded from probe design									
2c		2	Excluded from probe design									
**3**	**Regulatory repeats only (no intergenic/intronic, no coding repeats)**	**390**	**280**	**228**	**188**	**101**	**110**	**+ 30**	**+ 20**	**+ 5**	**+ 0**	**- 30**
**4**	**Additional regulatory repeats within 1 kb from the genes involved in XLID (not yet included)**	**68**	**62**	**55**	**51**	**37**	**6**	**+ 3**	**+ 8**	**+ 7**	**+ 2**	**- 3**
	Total ("functional")	1007	715	611	515	308	270	+ 66	+ 49	+ 15	+ 2	- 66
**5**	**Intronic repeats only**	**3431**	**1974**	**1351**	**826** (682 with 30%≥GC≥70%)	**56**	**1457**	Excluded from further probe design				
**6**	**Intergenic repeats only**	**4126**	**1814**	**1120**	**645** (521 with 30%≥GC≥70%)	**34**	**2312**	Excluded from further probe design				
	Total ("non-functional")	7557	3788	2471	1471	90	3769					
	Total (all)	8564	4520	3096	1997	402	4044	+ 66	+ 49	+ 15	+ 2	- 66

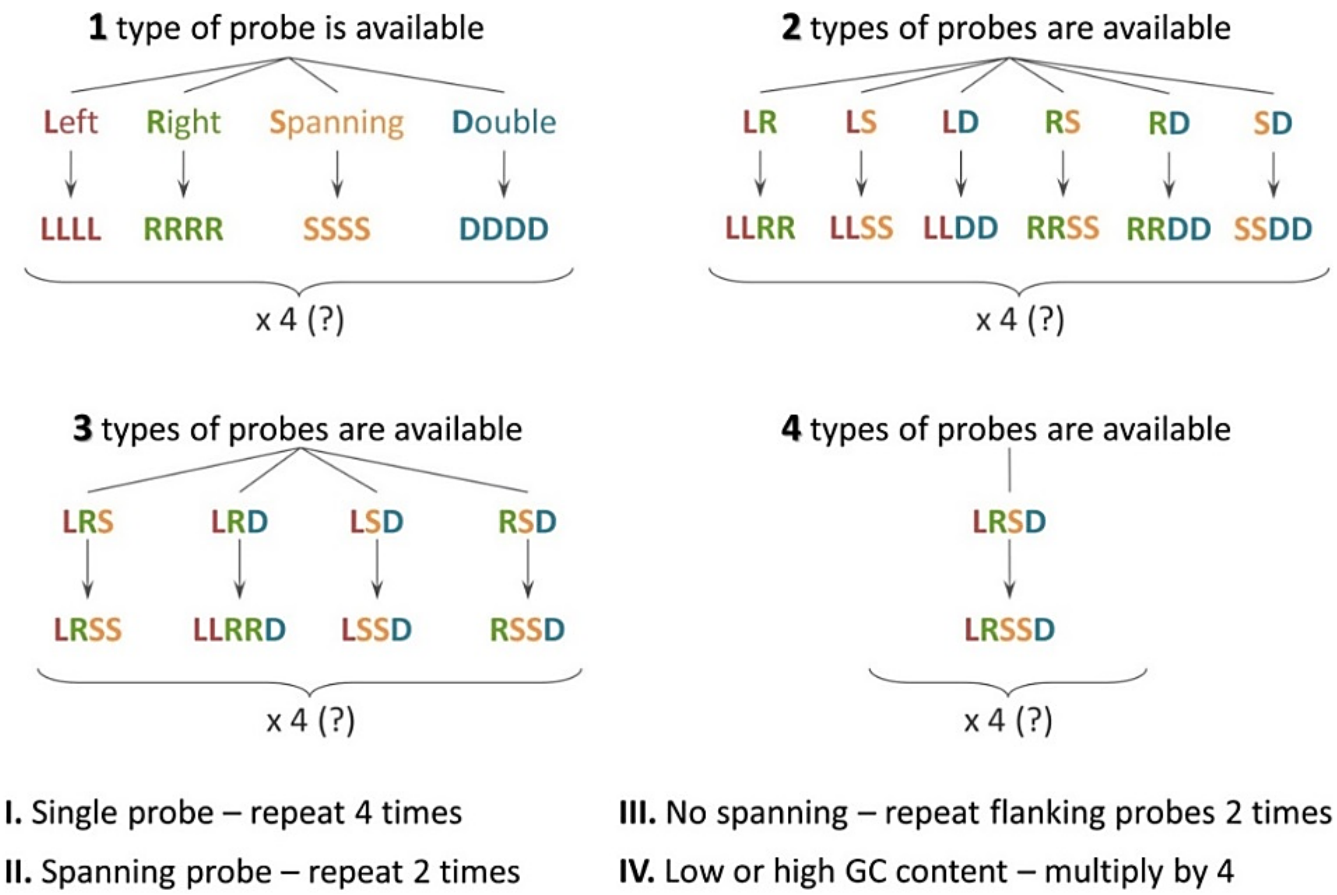

Figure S1 Scheme for capture probe multiplication in cases when 1, 2, 3 or 4 types of unique probes are available, and possible combinations of these types: L – left flanking, R – right flanking, S – spanning, D – double (or special) probe. Main principles of the algorithm for probe multiplication are mentioned at the bottom (I-IV)

Additional file 2 Sample table of the capture probes (for the full table see Zablotskaya_Additional_file_2.xlsx)

# File Type: SureSelect DNA Probe Upload Report			
# Timestamp: 06-Oct-2015			
# User: alena.zablotskaya@kuleuven.be			
# Created By: SureSelect DNA Advanced Design Wizard -- Probe Upload			
# Probegroup Name: TandRep_chrX_KULeuven_20151005_1			
# Species: H. sapiens (H. sapiens, hg19, GRCh37, February 2009)			
#			
# Columns:			
# TargetID: Identifier of the target of the probe.			
# ProbeID: Identifier of the probe.			
# ProbeSequence: Sequence of the probe.			
# ReplicationCount: The number of times this probe appears in the probe group.			
#			
# Probe Summary			
# Total Probes: 5486			
# Duplicate Probes Removed: 0			
# Probes Not Uploaded: 0			
# Existing Probes Updated: 5486			
# New Probes: 0			
# Probegroup Size: 493.000 kbp			
#			
# Probe Upload Parameters			
# File Name: SureSelect_FinalProbes_replicated2.zip			
# Probe Precedence: Overwrite			
# Boosting: UserProvided			
TargetID	ProbeID	Sequence	Replication
chrX_228962_229161_229142_229261_R	Pchrx_1	GGTATAGACAGGGTGGTAAAGCTCTGGATGGGAGGATGGGGTAGATGACATCCTCACCTTGGCTGCTGTACCCCACACAGAGACCACAGGAAACCCGTCTCCTGGGTGAGCTCTCACAGC	4
chrX_299255_299352_299155_299274_L	Pchrx_2	CGGCTGTCCTTACCTCACCGGCCCCGTGTCAGGTCTCACCCCGCCCCTCTGTGTCCGCGCACCCCACCTGACTGACCCCGCATCCCCCGGCTGCACACACACCCGTCCTCCCACTCACCC	10
chrX_299255_299352_299333_299452_R	Pchrx_3	ACTCACCCGTCCTCCCAGGGCTCTCCCGCAGTCTCCTCGGCCACCAGGATGTCGTACTCCTCGGCCGCGTACTTCTCCCAGTCCGAGAGCTCGGGGCCGCCGCTGTCACCGTCCTGGAGG	2
chrX_299255_299352_299195_299412_D	Pchrx_4	CCGCCCCTCTGTGTCCGCGCACCCCACCTGACTGACCCCGCATCCCCCGGCTGCACACACCTCTCCCGCAGTCTCCTCGGCCACCAGGATGTCGTACTCCTCGGCCGCGTACTTCTCCCA	1
chrX_306087_306160_305987_306106_L	Pchrx_5	GCTTCAGGACCAGCGGCCCACACTGACCCTGTAGACGCCCCCAGGCAGAGGCCTCGGGCAGAGGCGCACGCGGGGACCCAGACACGGGGCAGCGACTGGGGAAGGAGAGGCAGCTGCAGA	10
chrX_306087_306160_306141_306260_R	Pchrx_6	AGACACAGAGCTGGGAGTGCGGAAGGAGAGGCAGCTGCAGACCCGCAGGCCCTGAGAGAAGGGTGTGCTCGGTGTCCGCGCGGCCCGCCCGCCCCTGTGCCCCCACGCACCGTGGTCATT	2
chrX_306087_306160_306027_306220_D	Pchrx_7	CCAGGCAGAGGCCTCGGGCAGAGGCGCACGCGGGGACCCAGACACGGGGCAGCGACTGGGGGAAGGAGAGGCAGCTGCAGACCCGCAGGCCCTGAGAGAAGGGTGTGCTCGGTGTCCGCG	5
chrX_323932_324395_323832_323951_L	Pchrx_8	GCTTCCCGCAGTAGCTGCCGACTGAGTGCTCCTTCAGCACAGCTTTACCTAAGGGCACACCCCTTCCCTGTGATTCAGAACGTGACCTCTGTGTCCTGGGGCAGGAGGTCCCTGCATCTC	2
chrX_323932_324395_324376_324495_R	Pchrx_9	CAGTCATCTCCCTGTGCCCACCATCCCACAATGCATCTCCCCAAGCCCGCGATCCCACAGTGCATCTTCCCGAGCCCACAACGCATTCCCGCAGCGGCGCCTTCCCGTCTCTCGCTGTGG	2
chrX_323932_324395_323872_324455_D	Pchrx_10	AGCTTTACCTAAGGGCACACCCCTTCCCTGTGATTCAGAACGTGACCTCTGTGTCCTGGGCCATCCCACAATGCATCTCCCCAAGCCCGCGATCCCACAGTGCATCTTCCCGAGCCCACA	1
chrX_336039_336374_335939_336058_L	Pchrx_11	CCTCTGACCCCCGGTAAGTCTCCAAAGGGAAGCTTTAAGACAGCACCAGAGGGAATGTGCTCAAACCACCAGGAGACGATTTAAACAAGTGCACCCAGTAACCCACGATGCGGGGCGCAA	2
chrX_336039_336374_336355_336474_R	Pchrx_12	GGCAGAGGTGCCGCCCCTTCAGTTCATATCAGCACTACACACGGACGGCCTCCCAAACGCAGCTAGGGGAACCAGATTGGGAGCCAACCTCCTGTGATCTCCCTCCGAAAGTCAAAGATC	2
chrX_336039_336374_335979_336434_D	Pchrx_13	CAGCACCAGAGGGAATGTGCTCAAACCACCAGGAGACGATTTAAACAAGTGCACCCAGTAAGTTCATATCAGCACTACACACGGACGGCCTCCCAAACGCAGCTAGGGGAACCAGATTGG	1
chrX_605105_605200_605005_605124_L	Pchrx_14	GGAGAAGGGGCGACGCTCCCTAGGGGAGAAGAGGCACGTTGGAGGTTTCCGGGGGCGCGGGGCGGAGCAGGCCCCCCAGTCCCCATCCTGCGCCCTCACCCCGCCGGGTCCGCTCCCGCA	10
chrX_605105_605200_605045_605260_D	Pchrx_15	GGAGGTTTCCGGGGGCGCGGGGCGGAGCAGGCCCCCCAGTCCCCATCCTGCGCCCTCACCCCCTACCTGATGTTCCCCCCGCCGCCCTTCGGGCTGCCCATCGCGTCGCTGGCCGAGTCC	10
chrX_605259_605294_605275_605394_R	Pchrx_16	CGTGGTCGCCGCCGCCGCCAAAAGCAACAGCAAGAATTCCAGCATCGCCGACCTGCGGCTCAAGGCGCGGAAGCACGCGGAGGCCCTGGGGCTCTGACCCGCCGCGCAGCCCCCCGCGCG	10
chrX_605259_605294_605199_605354_D	Pchrx_17	CGCCCTACCTGATGTTCCCCCCGCCGCCCTTCGGGCTGCCCATCGCGTCGCTGGCCGAGTAAAGCAACAGCAAGAATTCCAGCATCGCCGACCTGCGGCTCAAGGCGCGGAAGCACGCGG	2
chrX_620193_620228_620093_620212_L	Pchrx_18	ATTACAAGCATGAACCACTGCCCGTGGTCTCCAAAAAAAGGACTGTTACGTGGATGTTCTAGCTTCCTGTTCTCGTCTTTTCTTTGTTAATTGTACAGTTTGAGGGTGTGTGTGCGTGTG	2
chrX_620193_620228_620209_620328_R	Pchrx_19	TGTGCGCGCGTGTGTGTGTGCAGTCTCCTGATTTCATGTATTTAATTGTTATTACCACCACCTCCATCTCTCATTCCTTCTTACCCTCACTGTGTAAAGATACATGTTGTTTTTAAATTT	6

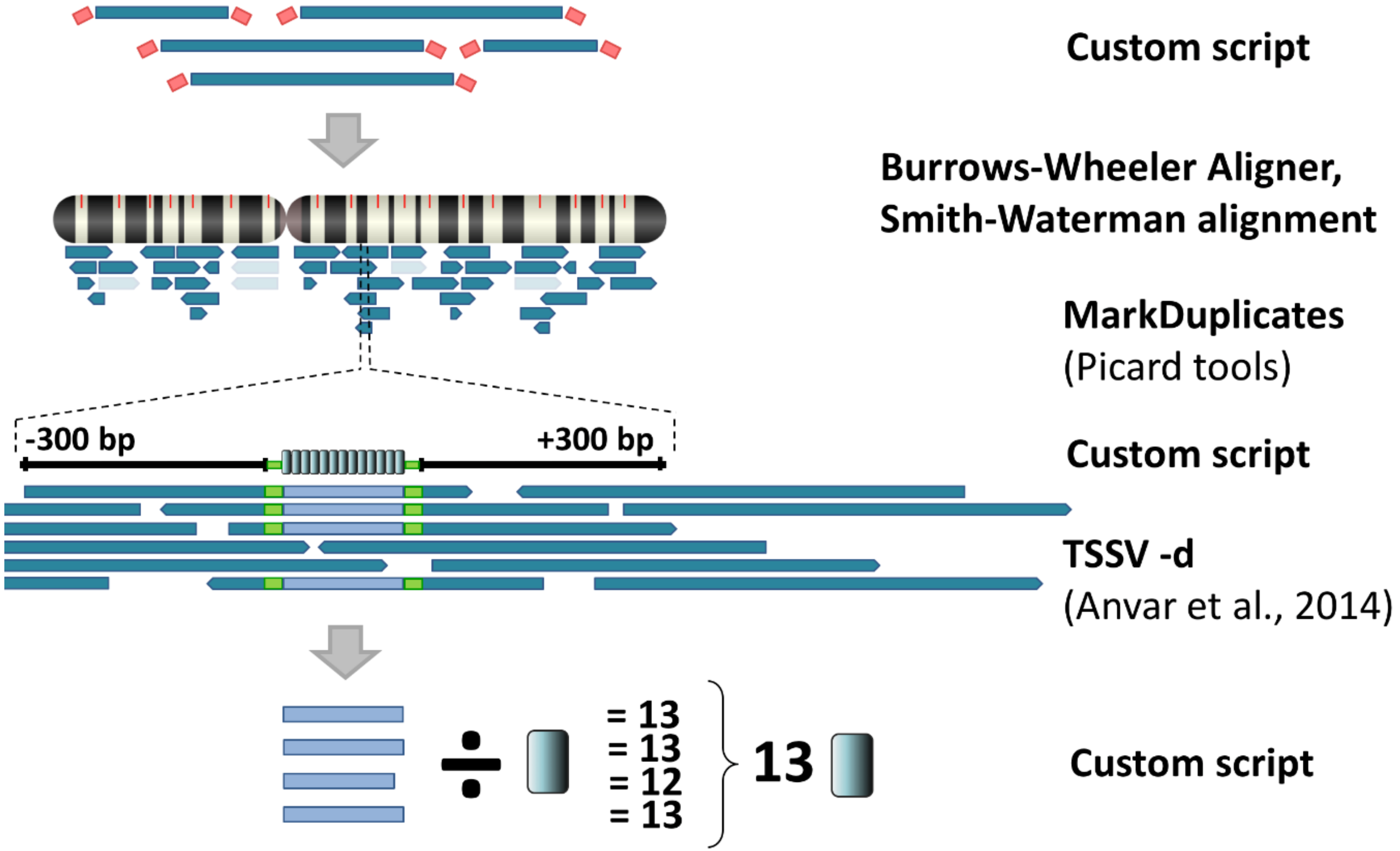

Figure S2 Scheme for bioinformatical analysis of tandem repeats in sequencing data

Additional file 9 Sample table of the PCR primers (for the full table see Zablotskaya_Additional_file_9.xlsx)

Family	Marker code	Tandem repeat characteristics											M13 tail -	ACGTTGTAAAACGACGGCCAG		
		Chr	Start	End	Score	Unit length	Copies	Purity	Sequence (unit)	Repeat length	SERV score	Annotation	Estim. amplicon size	M13 tail-Forward primer sequence	Reverse primer sequence	T_{anne}
L020	XLID1	chrX	41377018	41377074	52	3	19	98,2	AGC	57	1,039	ThreePrimeUTR	185	ACGTTGTAAAACGACGGCCAGTCTGCTGATGGAATGGAA	ATGACCTAGCTGCATATAC	54
	XLID2	chrX	44007461	44007502	32	2	21	90,5	GT	42	1,008	ThreePrimeUTR	333	ACGTTGTAAAACGACGGCCAGGGGATGTGTGTGTAAAAC	CAACCCACCTCATCAAAA	54
	XLID4	chrX	41333072	41333368	28	33	9	60,3	CCTCGGCCTCCTGGGCCTCCGCGCCCTCGCCGC	297	-0,384	Coding	436	ACGTTGTAAAACGACGGCCAGCCACAACAACCTGTCCTT	GTCGTTGAGCAGCAGATG	57
	XLID5	chrX	41333415	41333666	32	36	7	63,5	CTCTACGGCAACCTGCTGGCCCTCCTCGCGCTCGAC	252	-0,648	Coding	727	ACGTTGTAAAACGACGGCCAGCCACAACAACCTGTCCTT	GTTGCGGAAGAGGAAGAG	57
	XLID6	chrX	42637031	42637084	23	27	2	96,3	TCCTTCGACATCGCGCACTGAATCTTC	54	-0,933	Coding	267	ACGTTGTAAAACGACGGCCAGCGCTTTTTCTCCTGTTTGT	GTGGGACGAATCAAGCAT	56
	XLID7	chrX	42636638	42636735	39	49	2	94,9	TTCACAAACAATTGCAAGGGTGAAGCAGGCATCTTCTAAGGGTCGCATG	98	1,133	ThreePrimeUTR	222	ACGTTGTAAAACGACGGCCAGATGCGCTACAGAAAACAAGA	AGGCACGAACGAAGAGAA	57
	XLID8	chrX	46334046	46334093	24	4	12	79,2	ATTT	48	0,727	ThreePrimeUTR	299	ACGTTGTAAAACGACGGCCAGCATGGATAAGGGAGGGGT	GGCTGAGGTGGGAGGATAA	57
	XLID9	chrX	46357365	46357430	20	22	3	81,8	TATATGAATACAAATTTATACT	66	-0,621	ThreePrimeUTR	122	ACGTTGTAAAACGACGGCCAGATACACACACACTCACCT	CTGGGTGGTTGACATATAAA	55
	XLID10	chrX	43741611	43741650	22	4	10	82,5	CCTG	40	0,580	FivePrimeUTR	213	ACGTTGTAAAACGACGGCCAGGACTAACCTGAGATGCCG	GGGGCTGGTAATATAGCG	55
	XLID11	chrX	43832704	43832765	48	2	31	90,3	AG	62	0,902	FivePrimeUTR	192	ACGTTGTAAAACGACGGCCAGCTTTTGTTCTTCTGTCCCTCT	CGCACGCCTGATTGATAT	57
	XLID12	chrX	44732597	44732680	29	3	28	69	CGC	84	0,533	FivePrimeUTR	315	ACGTTGTAAAACGACGGCCAGCTCCAACGAATCCCCTCA	ATTCACAACTCCAACGCC	58
	XLID13	chrX	43514345	43514452	72	6	18	86,1	CGGCAC	108	1,115	CpGIslands	431	ACGTTGTAAAACGACGGCCAGGGTACCGAGAACAGCCTGA	TGGGGAGTGTATGCTGGA	58
	XLID14	chrX	43515121	43515204	22	42	2	88,1	GCCACAAGCACCTCCTGCACCCCATAACATCCCCCCAGTGTC	84	-0,394	Upstream	295	ACGTTGTAAAACGACGGCCAGACCTCCTATACCCAATGAC	GCAGCTGGAAAGGATTAC	55
	XLID16	chrX	41782416	41782487	20	6	12	68,1	GCCGGC	72	0,480	CpGIslands	656	CCGGGGCATCACTGAAAT	ACGTTGTAAAACGACGGCCAGGGAGGAGGAGAAAGAGGAGG	59
	XLID17	chrX	42637497	42637652	62	6	26	71,8	TACCGC	156	1,043	Upstream	351	ACGTTGTAAAACGACGGCCAGATTTTGCGGAGAGAAGGG	AAAAATGACGTTAGCATGGG	57
	XLID20	chrX	45046714	45046751	36	2	19	100	CA	38	1,008	oregannoTFBS	213	ACGTTGTAAAACGACGGCCAGGGCTGTGTTTCTGTTTGT	AAGACGACAGGACCAAGA	56
	XLID22	chrX	45386687	45386738	50	2	26	100	CA	52	0,809	Downstream	121	ACGTTGTAAAACGACGGCCAGAGATGGAAATCAGGGGAG	GTATGAGGCAAAGAAGATGAG	56
	XLID23	chrX	45605926	45606021	42	4	24	74	TGGA	96	0,965	Upstream	348	ACGTTGTAAAACGACGGCCAGTGGCATTTGTCTTTTCTACC	TTTTTTTCTCTTTCCCCCCT	55
	XLID24	chrX	45606026	45606065	20	4	10	80	GATA	40	0,506	Upstream	184	ACGTTGTAAAACGACGGCCAGTGGATGGATGGATGGGTG	GGGGGAGGAATCTGGATA	56
	XLID25	chrX	45606270	45606355	48	2	43	79,1	GT	86	1,062	Downstream	318	ACGTTGTAAAACGACGGCCAGGGGGGGAAAGAGAAAAAAAGA	TGAGAGAGATTATTGGGCAGT	56
	XLID26	chrX	45707349	45707393	26	3	15	82,2	AAC	45	0,968	NonCoding	194	ACGTTGTAAAACGACGGCCAGAGGTGAAGGTTGCAGTGA	AGAAGACGCCAAAGGGAA	57
	XLID27	chrX	45709592	45709631	38	2	20	100	GT	40	0,970	NonCoding	402	ACGTTGTAAAACGACGGCCAGATGAATGACTGCGGAAGG	TGGTGCTTGTGTCTTTGG	57
	XLID28	chrX	42638446	42638471	51	1	30	93	A	30	0,867	Upstream	198	ACGTTGTAAAACGACGGCCAGTTCTTCCCACTCTACCTCC	TATTTGCTGTTGGGGGTC	55
	XLID39	chrX	41746550	41746595	44	2	23	100	CA	46	0,862	Intron	442	ACGTTGTAAAACGACGGCCAGACAACCTTCAAAATCAGCCACA	TCCCTTCTACTCATGCACACT	59
	XLID40	chrX	43178073	43178106	32	2	17	100	CA	34	1,073	Intergenic	385	ACGTTGTAAAACGACGGCCAGTAGAAGTCAAGGTGGGGCTG	TCAACTCTGAGGGGCCATTT	59
	XLID41	chrX	43897422	43897457	34	2	18	100	AC	36	1,044	Intergenic	313	ACGTTGTAAAACGACGGCCAGTTTTGCATGGAGAGTGTGGC	GATGCAATCTTTCCGGCCTC	59
	XLID42	chrX	80492355	80492432	42	2	39	78,2	AG	78	1,057	Intron	263	ACGTTGTAAAACGACGGCCAGTGGCAGTGTTTGGAATTAGCC	TCCTTGCCACTTAGAAGCCA	59
	XLID43	chrX	80682785	80682818	32	2	17	100	GT	34	1,073	Intergenic	375	ACGTTGTAAAACGACGGCCAGTGGCAGTCAGTTGGTGGAAT	TGCAAGGGAAAGAAAACTACCC	59
	XLID44	chrX	83134725	83134770	28	2	23	82,6	AC	46	1,009	Intron	214	ACGTTGTAAAACGACGGCCAGCCACTTTCACCCATCGCTTT	CCCAGCTGCCATACACTTTC	59
	XLID45	chrX	84768208	84768237	28	2	15	100	TG	30	1,103	Intergenic	333	ACGTTGTAAAACGACGGCCAGGGGCATCCTCAAATCAGATAGC	CATGTCTAGCCCCTTCCTCC	59
	XLID46	chrX	85757000	85757075	64	4	19	94,7	TCCT	76	1,065	Intron	487	ACGTTGTAAAACGACGGCCAGACCAGTTAGAGCGGGGTTAA	CCCTAACACCCTTTCCAGCT	59
	XLID47	chrX	86914941	86914998	38	2	29	84,5	TG	58	0,958	Intron	368	ACGTTGTAAAACGACGGCCAGGTCATGTTCTGTGCCTGAGG	TGAGTTGTGTGCTGTCTATGAC	58
	XLID48	chrX	88396020	88396053	30	2	17	97,1	CA	34	1,075	Intergenic	238	ACGTTGTAAAACGACGGCCAGTCTTTGGAGGGGAAGGAGTATG	TGTGGCTAAGCTGTAATTTCCTG	59
	XLID49	chrX	96030353	96030390	36	2	19	100	TG	38	1,008	Intron	192	ACGTTGTAAAACGACGGCCAGGAGTGTCTACAGTTTGGGGC	ACCATCAATAGAAGAGGCACCA	59
	XLID50	chrX	42325649	42325680	30	2	16	100	AC	32	1,094	Intergenic	355	ACGTTGTAAAACGACGGCCAGGCACTTAAAGAGCTGGGATCA	GACATCCTGCCTAGTTCCCA	58
	XLID51	chrX	42505901	42505938	36	2	19	100	TG	38	1,008	Intergenic	328	ACGTTGTAAAACGACGGCCAGAGAACTATGCAAACAATGGGGA	GGGATTCTCTGCCCTCTGTT	59
	XLID52	chrX	87004917	87004948	30	2	16	100	AC	32	1,094	Intergenic	453	ACGTTGTAAAACGACGGCCAGTTACGAGAGACTCTGAGACCT	AAAATGAGACACAAAAGGCAGAA	57
	XLID61	chrX	49821981	49822370	342	26	15	97,2	TCCTGGATAGATACTATCCAGGACTC	390	1,453	Intron	501	ACGTTGTAAAACGACGGCCAGTGCCCTGAATAAGTGTCCCA	CCAGTGGCCAAGAAGCAAAT	59
L061	XLID72	chrX	66765149	66765262	77	3	38	85,1	AGC	114	1,025	Coding	369	ACGTTGTAAAACGACGGCCAGATGGAAGTGCAGTTAGGGCT	AGGTTGCTGTTCCTCATCCA	59
	XLID73	chrX	70151351	70151390	38	2	20	100	GT	40	0,970	Upstream	229	ACGTTGTAAAACGACGGCCAGAAGGTAGGTGGGGTGTGATG	GCCTGCATTAGAAACACCCA	59
	XLID74	chrX	74743332	74743375	42	2	22	100	AC	44	0,894	NonCoding	232	ACGTTGTAAAACGACGGCCAGGACCTCCGCAAAATCGTGAA	ATGGCTCGCATCTTTCACAC	59
	XLID75	chrX	77266068	77266093	52	1	26	100	T	26	0,770	ThreePrimeUTR	389	ACGTTGTAAAACGACGGCCAGGCATTTCCCCTAGCAGTTGG	AGCTGGAAATAGTGGCAGGT	59
	XLID76	chrX	84343323	84343351	58	1	29	100	T	29	0,807	NonCoding	355	ACGTTGTAAAACGACGGCCAGAGAATGGTGTATGGAAAGGTGT	AGGAGGCACAGTCAACTTTG	58
	XLID77	chrX	84363558	84363947	151	15	26	71,3	CATGCCTGGTTGGCT	390	1,390	Coding	481	ACGTTGTAAAACGACGGCCAGTTGTCTCATGCTTGGTTGGC	CCAATCAGGCACGAACCAAT	59
	XLID78	chrX	84499126	84499197	47	3	24	84,7	CGG	72	0,999	FivePrimeUTR	458	ACGTTGTAAAACGACGGCCAGAACCCGAATGTGAGGACCTT	TACCGTCACCCTAAACCACC	59
	XLID79	chrX	102630531	102630560	60	1	30	100	T	30	0,842	Upstream	359	ACGTTGTAAAACGACGGCCAGCTTGATGTTGTGTGGAGCCC	TGAGGCCAGTAGTGTGAGAC	59
	XLID80	chrX	106184602	106184641	38	2	20	100	GA	40	0,970	ThreePrimeUTR	306	ACGTTGTAAAACGACGGCCAGAAACTGGCCTCTTTCTCCAC	CGTGAGATCGGGTATGACTCA	58
	XLID81	chrX	47097175	47097264	50	2	45	78,9	GT	90	1,046	Intron	188	ACGTTGTAAAACGACGGCCAGAGGCTGCTCAGTATTTGGGA	GCTTGGACTTCAGGACTTGG	59
	XLID82	chrX	51887044	51887123	54	2	40	85	CA	80	1,076	Intergenic	322	ACGTTGTAAAACGACGGCCAGCCATGTGGGCAGGTTTTGAT	TTAATACAGCTGGGGAGGGG	59
	XLID83	chrX	116923786	116923815	28	2	15	100	GT	30	1,103	Intergenic	294	ACGTTGTAAAACGACGGCCAGCCCTCTATCTGCCTGCTCTG	CAGAGTGGCTAGGTCCCTTT	59
	XLID84	chrX	119421162	119421191	26	2	15	96,7	AC	30	1,089	Intron	396	ACGTTGTAAAACGACGGCCAGTGACCATGGCCTCATTAGCA	AACCCAATCTACTGCCTGCC	60
	XLID85	chrX	46532781	46532818	26	2	19	86,8	AC	38	1,053	Intron	411	ACGTTGTAAAACGACGGCCAGTCATGGAACCTGAAGCGTCT	GAGGAGGCAGAACTGTGTCT	59
	XLID86	chrX	119746516	119746567	48	2	26	98,1	GT	52	0,821	ThreePrimeUTR	452	ACGTTGTAAAACGACGGCCAGGGTAGACCTCTAGAGAAACTGTC	AGATCGTACCACTGCACTCC	58
	XLID43	chrX	80682785	80682818	32	2	17	100	GT	34	1,073	Intergenic	375	ACGTTGTAAAACGACGGCCAGTGGCAGTCAGTTGGTGGAAT	TGCAAGGGAAAGAAAACTACCC	59
	XLID45	chrX	84768208	84768237	28	2	15	100	TG	30	1,103	Intergenic	333	ACGTTGTAAAACGACGGCCAGGGGCATCCTCAAATCAGATAGC	CATGTCTAGCCCCTTCCTCC	59
	XLID46	chrX	85757000	85757075	64	4	19	94,7	TCCT	76	1,065	Intron	487	ACGTTGTAAAACGACGGCCAGACCAGTTAGAGCGGGGTTAA	CCCTAACACCCTTTCCAGCT	59

Table S2 Primers used for qPCR

Gene	Primer pair	Primer direction	Primer sequence
FMR1	FMR1_1	Forward	AAA ATG TTC CAC AAG AAG AGG AAA TT
		Reverse	GCA TTA GGT CCA ACC CTT GAA T
PORCN	PORCN_1	Forward	CTC TGC CGA CAT TCC TCC
		Reverse	TGT GCA TCT CAC CCA TGA GT
FMR1	FMR1_2	Forward	CGC ACG GGT AAA GAT CGT A
		Reverse	TCA CGA GTG GTT GCT GAC
HPRT	HPRT_1	Forward	TGA CAC TGG CAA AAC AAT GCA
		Reverse	GGT CCT TTT CAC CAG CAA GCT
GUSB	GUSB_1	Forward	AGA GTG GTG CTG AGG ATT GG
		Reverse	CCC TCA TGC TCT AGC GTG TC
FMR1	FMR1_3	Forward	AGT TGT GAG GGT GAG GAT
		Reverse	TGG AAG GAA GGG AAT TTG GT
ADAM23	ADAM23_1	Forward	CCA ACA AAG CTA TTT GAG CCC
		Reverse	CCA CAA TCA CAC TCC TCC C
PEG10	PEG10_1	Forward	CTG TCT TCG CAG AGG AGT C
		Reverse	CCC TCT TCC ACT CCT TCT TT
NRCAM	NRCAM_1	Forward	AAT AAC ATT GTT GTC CGC CC
		Reverse	TTG AAG TGT GAT TGG TTC AAG TTT
OAT	OAT_1	Forward	CTG ATG TTG TAA CTG CCG T
		Reverse	CAA GCA TCC CAA TCT TTG GT
PIEZO2	PIEZO2_1	Forward	GCG ATG GCC TCA GAA GT
		Reverse	CAT TGT ATC GGA ATG CAC ATG CTA
NEFH	NEFH_1	Forward	CAG GAC CTG CTC AAT GTC A
		Reverse	CTC TTC ACC TTC CAG GAG TT
PLXNB2	PLXNB2_1	Forward	CTC AAG GTG TAC CTC ACC C
		Reverse	CTC TTG TTT ATC TCC ACA AGG ATA G
ARMCX2	ARMCX2_1	Forward	TCT AAC TGA ACT GAA GTA AGG AGA AAC A
		Reverse	CAG TTG CAC AGA GCC CA
FOXO3	FOXO3_1	Forward	CTC ATC TCA GAG CTG GGT G
		Reverse	TCA GTT TGA GGG TCT GCT TT

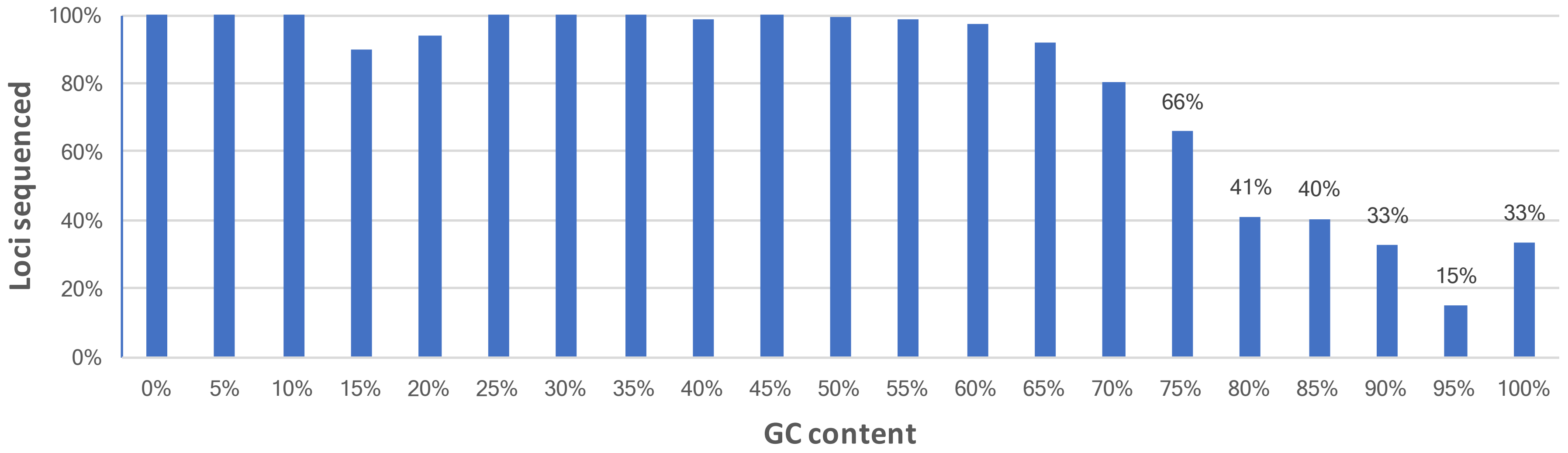

Figure S3 Portion of successfully sequenced tandem repeats drops to 15-40% for GC-rich loci

Additional file 10 Sample table of genotyped TRs (for the full table see Zablotskaya_Additional_file_10.xlsx)

													5X20 sample	Allele_1		Allele_2	
chrom	chromStart	chromEnd	name	Score	Unit le	Copie	Purity	Sequence (unit)	Repeat	SERV Score	Annotation	G+C %	Total CCSs	Copies	Supp.rea	Copies	Supp.read
chrX	228962	229161	etandem	70	40	5	77,5	GTTGATGTAGACGGGAGGATGGTATAGACAGGGTGGTGGT	200	-0,231099374	Coding	50,75%	1	5	1		
chrX	299255	299352	etandem	43	49	2	96,9	ACCCGTCCCCCACTCACCCGTCCTCCCCACTCACCCGTCCTCCCAGGG	98	1,398768567	CpGIslands	72,16%	8	2	8		
chrX	306087	306160	etandem	33	37	2	97,3	GAAGGAGAGGCAGCTGCAGACACAGAGCTGGGAGTGC	74	-0,102527825	Coding	63,01%	3	0	2	2	1
chrX	323932	324395	etandem	329	29	16	88,6	ACGATCCCACAGTGCATCTCCCCGAGCCC	464	1,431552363	CpGIslands	65,23%	12	1	4	11	3
chrX	336039	336374	etandem	252	48	7	94,6	ACCCACGATGCGGGGCGCAAACTCACAGGGCAGAGGTGCCGCCCAGTC	336	2,770232259	CpGIslands	65,07%	4	7	4		
chrX	605105	605200	etandem	20	6	16	63,5	CACCCG	96	0,429532082	Coding	77,89%	0				
chrX	605259	605294	etandem	21	3	12	83,3	CCG	36	0,788413846	Coding	88,57%	0				
chrX	620193	620228	etandem	22	2	18	83,3	TG	36	1,022338533	Downstream	65,71%	5	18	5		
chrX	1331700	1331731	microsatellite	30	2	16	100	AC	32	1,094349439	Upstream	51,61%	0				
chrX	1572563	1573018	etandem	300	38	12	87,1	GGGAGGGGCGTGGAGATGGGGGTGTCCACGGGCCTGGT	456	2,03662518	FivePrimeUTR	72,31%	0				
chrX	1718143	1718286	etandem	35	3	48	63,2	CAG	144	0,447494208	Coding	61,54%	2	48	2		
chrX	1719564	1719698	etandem	38	27	5	74,1	CGGGGGCTGCAGCAGAAGGAGCGGGAG	135	-0,376854458	Coding	70,90%	0				
chrX	1720192	1720401	etandem	28	42	5	66,7	GGCACGACCGGAAGCGGAGCCGGGAGCGGCGGGGCAGCACCA	210	-0,843079039	Coding	73,68%	0				
chrX	2583202	2583289	etandem	62	4	22	87,5	CCTT	88	1,015914385	NonCoding	50,57%	8	41	3	22	2
chrX	2746152	2746207	etandem	30	4	14	80,4	TCAT	56	0,921111889	Upstream	41,82%	19	14	18	15	1
chrX	2746294	2746361	etandem	24	4	17	70,6	TCAT	68	0,729838664	Upstream	40,30%	17	17	16	16	1
chrX	2746508	2746547	etandem	22	4	10	82,5	TCAT	40	0,580375988	Upstream	33,33%	15	10	15		
chrX	2779577	2779675	etandem	32	33	3	82,8	TGTGGGCGTGCCGTGTGCAAATTCACCACCGGG	99	-0,718692356	Coding	61,22%	5	3	5		
chrX	2798457	2798508	etandem	32	2	26	82,7	GT	52	0,98688029	Intron	50,98%	17	26	12	25	3
chrX	3030577	3030676	etandem	42	50	2	96	GGTCCTAACGAGAAGAGATAATTACAATCAGGCTACCAAAGGAAGCACTA	100	1,389572917	ThreePrimeUTR	42,42%	18	2	18		
chrX	3190147	3190180	etandem	28	2	17	94,1	CA	34	1,079264725	Intergenic	42,42%	11	17	9	18	2
chrX	3312273	3312308	etandem	30	2	18	94,4	GT	36	1,061554636	Intergenic	45,71%	21	18	20	17	1
chrX	3467394	3467423	etandem	26	2	15	96,7	CT	30	1,089199824	Intergenic	44,83%	16	20	7	19	5
chrX	3631196	3631387	etandem	20	48	4	67,7	GCGGGGGCCGCCCTCCGGCGCCGCGGCCTGGGCCAGCCCCGCGGCCTC	192	-0,880317262	Coding	81,68%	0				
chrX	3951794	3951825	microsatellite	30	2	16	100	TG	32	1,094349439	Intergenic	51,61%	15	16	9	15	5
chrX	4111544	4111855	etandem	181	3	104	79,5	TGA	312	1,166751661	Intergenic	40,19%	14	104	12	100	1
chrX	4394119	4394150	microsatellite	30	2	16	100	TG	32	1,094349439	Intergenic	51,61%	16	16	13	15	2
chrX	4435040	4435075	microsatellite	34	2	18	100	CA	36	1,04375093	Intergenic	48,57%	7	18	5	16	1
chrX	4559423	4559494	etandem	42	2	36	80,6	TC	72	1,038135922	Intergenic	46,48%	19	36	17	37	1
chrX	4679781	4679910	etandem	46	2	65	68,5	AG	130	1,181881818	Intergenic	48,84%	21	67	15	66	3
chrX	4701563	4701592	etandem	22	2	15	90	AG	30	1,052306102	Intergenic	58,62%	22	15	14	14	6
chrX	4904608	4904639	etandem	26	2	16	93,8	TG	32	1,085401427	Intergenic	45,16%	7	16	4	15	2
chrX	4941605	4941900	etandem	178	4	74	80,7	CCAT	296	1,280934969	Intergenic	43,39%	19	74	18	72	1
chrX	5243260	5243305	etandem	30	2	23	84,8	CA	46	1,005440283	Intergenic	46,67%	15	23	14	22	1
chrX	5477218	5477251	microsatellite	32	2	17	100	TG	34	1,073475001	Intergenic	51,52%	5	17	2	18	2
chrX	5507875	5507908	etandem	24	2	17	88,2	AC	34	1,064627101	Intergenic	51,52%	9	18	6	19	1
chrX	5695969	5696000	etandem	26	2	16	93,8	CA	32	1,085401427	Intergenic	54,84%	28	16	18	15	8
chrX	5760188	5760331	etandem	51	3	48	68,8	AGG	144	1,014367349	Intergenic	44,76%	31	48	30	47	1
chrX	5808269	5808322	etandem	21	9	6	77,8	TTTTTTTAT	54	-0,086259278	ThreePrimeUTR	15,09%	14	6	13	5	1

Table S3 Screening of the chrX:45,606,270-45,606,355 repeat in controls by fragment analysis with the $(CT)_n$ and $(GT)_n$ sub-repeats differentiation

		Control unaffected males																			
	Proband	1	2	3	4	5	6	7	8	9	10	11	12	13	14	15	16	17	18	19	20
Full amplicon length, bp	**376**	372	338	372	374	354	366	366	372	366	372	372	366	364	372	374	366	372	372	372	366
Differential amplicon length, bp* (CT copy number)	273 (21)	265 (17)	253 (11)	265 (17)	265 (17)	265 (17)	265 (17)	265 (17)	265 (17)	265 (17)	265 (17)	271 (20)	265 (17)	265 (17)	265 (17)	265 (17)	265 (17)	265 (17)	265 (17)	265 (17)	265 (17)
Deduced GT copy number	(33)	(35)	(24)	(35)	(36)	(26)	(32)	(32)	(35)	(32)	(35)	(32)	(32)	(31)	(35)	(36)	(32)	(35)	(35)	(35)	(32)

* Obtained with a nested reverse primer TCACACACACACACAAAGAG

	Control unaffected males																			
	21	22	23	24	25	26	27	28	29	30	31	32	33	34	35	36	37	38	39	40
Full amplicon length, bp	370	370	338	370	370	370	366	370	370	368	372	372	374	368	364	368	362	372	374	366
Differential amplicon length, bp* (CT copy number)	265 (17)	265 (17)	253 (11)	265 (17)	265 (17)	261 (15)	265 (17)	265 (17)	265 (17)	265 (17)	265 (17)	265 (17)	265 (17)	265 (17)	265 (17)	265 (17)	265 (17)	265 (17)	273 (21)	265 (17)
Deduced GT copy number	(34)	(34)	(24)	(34)	(34)	(36)	(32)	(34)	(34)	(33)	(35)	(35)	(36)	(33)	(31)	(33)	(30)	(35)	(32)	(32)

* Obtained with a nested reverse primer TCACACACACACACAAAGAG

	Control unaffected males																			
	41	42	43	44	45	46	47	48	49	50	51	52	53	54	55	56	57	58	59	60
Full amplicon length, bp	370	368	372	372	354	362	370	366	370	354	372	362	368	374	372	368	**376**	358	372	370
Differential amplicon length, bp* (CT copy number)	265 (17)	265 (17)	265 (17)	265 (17)	265 (17)	259 (14)	265 (17)	265 (17)	265 (17)	265 (17)	265 (17)	261 (15)	265 (17)	265 (17)	265 (17)	265 (17)	265 (17)	261 (15)	265 (17)	265 (17)
Deduced GT copy number	(34)	(33)	(35)	(35)	(26)	(33)	(34)	(32)	(34)	(26)	(35)	(32)	(33)	(36)	(35)	(33)	(37)	(30)	(35)	(34)

* Obtained with a nested reverse primer TCACACACACACACAAAGAG

	Control unaffected males																			
	61	62	63	64	65	66	67	68	69	70	71	72	73	74	75	76	77	78	79	80
Full amplicon length, bp	366	372	364	372	366	338	338	372	370	372	372	362	374	338	354	366	370	368	338	372
Differential amplicon length, bp* (CT copy number)	265 (17)	265 (17)	265 (17)	265 (17)	265 (17)	253 (11)	253 (11)	265 (17)	265 (17)	265 (17)	265 (17)	259 (14)	271 (20)	253 (11)	265 (17)	265 (17)	265 (17)	265 (17)	253 (11)	267 (18)
Deduced GT copy number	(32)	(35)	(31)	(35)	(32)	(24)	(24)	(35)	(34)	(35)	(35)	(33)	(33)	(24)	(26)	(32)	(34)	(33)	(24)	(34)

* Obtained with a nested reverse primer TCACACACACACACAAAGAG

	Control unaffected males																			
	81	82	83	84	85	86	87	88	89	90	91	92	93	94	95	96	97	98	99	100
Full amplicon length, bp	372	340	374	366	372	374	354	354	372	372	354	340	354	354	372	372	374	370	**376**	370
Differential amplicon length, bp* (CT copy number)	265 (17)	253 (11)	271 (20)	265 (17)	265 (17)	269 (19)	265 (17)	265 (17)	265 (17)	265 (17)	265 (17)	253 (11)	265 (17)	265 (17)	265 (17)	265 (17)	265 (17)	265 (17)	271 (20)	265 (17)
Deduced GT copy number	(35)	(25)	(33)	(32)	(35)	(34)	(26)	(26)	(35)	(35)	(26)	(25)	(26)	(26)	(35)	(35)	(36)	(34)	(34)	(34)

* Obtained with a nested reverse primer TCACACACACACACAAAGAG
